# Kinetic modeling of Stickland reactions-coupled methanogenesis for a methanogenic culture

**DOI:** 10.1186/s13568-019-0803-8

**Published:** 2019-06-10

**Authors:** C. Sangavai, M. Bharathi, Shilpkar P. Ganesh, P. Chellapandi

**Affiliations:** 1 Molecular Systems Engineering Lab, Department of Bioinformatics, School of Life Sciences, Bharathidasan University, Tiruchirappalli, Tamil Nadu 620 024 India; 2 Department of Microbiology, Biogas Research Centre, Gujarat Vidyapith, Sadra, Gandhi Nagar, Gujarat 382 320 India

**Keywords:** Stickland reaction, Methanogenesis, Clostridium, Metabolic flux, Protein waste, Syntrophic degradation, Microbial mutualism, Glycine reductase

## Abstract

Studying amino acid catabolism-coupled methanogenesis is the important standpoints to decipher the metabolic behavior of a methanogenic culture. l-Glycine and l-alanine are acted as sole carbon and nitrogen sources for acidogenic bacteria. One amino acid is oxidized and another one is reduced for acetate production via pyruvate by oxidative deamination process in the Stickland reactions. Herein, we have developed a kinetic model for the Stickland reactions-coupled methanogenesis (SRCM) and simulated objectively to maximize the rate of methane production. We collected the metabolic information from enzyme kinetic parameters for amino acid catabolism of *Clostridium acetobutylicum* ATCC 824 and methanogenesis of *Methanosarcina acetivorans* C2A. The SRCM model of this study consisted of 18 reactions and 61 metabolites with enzyme kinetic parameters derived experimental data. The internal or external metabolic flux rate of this system found to control the acidogenesis and methanogenesis in a methanogenic culture. Using the SRCM model, flux distributions were calculated for each reaction and metabolite in order to maximize the methane production rate from the glycine–alanine pair. Results of this study, we demonstrated the metabolic behavior, metabolite pairing while mutually interact, and advantages of syntrophic metabolism of amino acid-directed methane production in a methanogenic starter culture.

**Electronic supplementary material:**

The online version of this article (10.1186/s13568-019-0803-8) contains supplementary material, which is available to authorized users.

## Introduction

The development of clean or environmentally friendly alternative energy is required to promote a global energy demand. Fossil fuels secure the demand, but they threat climate change due to the large burning. The development of sustainable energy resources is, therefore economically indispensable to recover a global energy need. Methane produced from methanogenic archaea inevitably a substitute for fossil fuels to satisfy such energy crises (Head and Gray 2016). A syntrophic association between acidogenic bacteria and methanogenic archaea is being of great concern to yield a large amount of methane in anaerobic digesters (Ali Shah et al. 2014; Enzmann et al. 2018). However, metabolic characteristics and mutualistic association of these trophic organisms are not yet known during the anaerobic digestion process. Organic wastes constituting carbohydrate, proteins, and lipids are gradually hydrolyzed and transformed into methane and CO_2_ by subsequent acidogenesis and methanogenesis. Generally, the methanogenic cultures exhibit a low potential to degrade protein-based waste compared to carbohydrate-based waste. Such limitation hinders the efficacy of the anaerobic digestion process and metabolic rate of methanogenic cultures.

Amino acids are used as the important carbon and energy sources for proteolytic microorganisms. Amino acid catabolic systems represent an advantage in protein-rich environments (Stark et al. 2017). Amino acids vary significantly in size and structure and are anaerobically or aerobically fermented via different pathways to a range of products depending on the type and concentration of amino acids present. These products include various organic compounds (mainly short-chain and branched-chain organic acids), ammonia, CO_2_ and small amounts of hydrogen and sulfur-containing compounds (Sangavai and Chellapandi 2017). The pairs of amino acids can be degraded through the Stickland reaction. Besides, a typical metabolic system may perform a single amino acid fermentation. The Stickland reaction usually involves one amino acid that acts as an electron donor (the product is shorter by one carbon atom than the original amino acid), while another acts as an electron acceptor (the product has the same number of carbon atoms as the original amino acid) (Stickland 1934; Nisman 1954). It occurs rapidly compared to uncoupled amino acid decomposition (Barker 1961). Certain amino acids can serve both as an electron donor and an electron acceptor (for example leucine). Therefore, the Stickland reactions are the simplest ways to ferment amino acids for microbial growth by providing approximately 0.5 mol ATP per mole amino acid transformed (Andreesen et al. 1989).

*Methanosarcina acetivorans* (MAC) is a heterotrophic methanogenic achaean that has a wide-substrate utility (Galagan et al. 2002; Nazem-Bokaee and Maranas 2018). *Clostridium acetobutylicum* (CAC) is an acidogenic bacterium and it has the ability to produce organic solvents and acids form protein catabolism (Sangavai and Chellapandi 2017). CAC and MAC shared interspecies electron transporter for being carried a consecutive flux of metabolites (Wang et al. 2011). Stickland reactions-coupled methanogenesis (SRCM) is a major mutualistic metabolic process occurring between them for complete anaerobic digestion of protein-based substrates for methane production. Metabolite distributions and flux coefficients of this system are not yet studied for methanogenic culture. CAC catabolizes one amino acid to acetic acid which in turn produces methane by MAC. A co-culture of *Clostridium collagenovoran* and *Methanosarcina barkeri* was extensively utilized for conversion of gelatin to methane (Jain and Zeikusi 1989). The specific methanogenic activity of mixed or developed methanogenic cultures on different protein-based substrates has been evaluated to reveal the SRCM (Chellapandi et al. 2008; 2010a; Chellapandi and Uma 2012a, b).

A kinetic model consists of a network structure, a corresponding set of rate expressions, and their associated parameter values. The size of kinetic models is ranging from single enzymes (Hattersley et al. 2011) and to entire pathways (Almquist et al. 2014; Costa et al. 2016; D’hoe et al. 2018; Kim et al. 2018). Metabolic modeling and simulation are currently advancing of mutualistic study for a better understanding of such a system (Chellapandi et al. 2010b). Several stoichiometric (Desai et al. 1999a, b; Ramasamy and Pullammanmappallil 2001) and kinetic models (Chellapandi 2011, 2013, 2015) have been formalized for studying the metabolic behaviors and methanogenesis of methanogens. A kinetic model has been developed for improved production of methane by a co-culture of *C. butyricum* and *M. mazei* (Bizukojc et al. 2010). Most recently, Ringemann et al. (2006) have explored the biochemical parameters as a selective pressure for gene selection that constitutes a metabolic pathway during inter-species and endosymbiotic lateral gene transfer. Hence, the present study was intended to develop a kinetic model for SRCM system consisting of CAC and MAC in a methanogenic culture and to perform a metabolic simulation for the production of methane from l-glycine and l-alanine as substrate constraints. This study would provide a new avenue to exploit protein-based waste as a substrate for methane production in batch digesters.

## Materials and methods

### Construction of the SRCM model

For the construction of SRCM model, we extracted information for the metabolic reactions, proteins, and genes from the genome-scale metabolic models of CAC and MAC (iMB745; iVS941; iMAC868) (Senger and Papoutsakis 2008a, b; Kumar et al. 2011; Benedict et al. 2012; Nazem-Bokaee et al. 2016). The missing enzymes involved in SRCM were identified by sequence similarity searching using NCBI-BLASTp program (Altschul et al. 1997). The functional equivalency of missing or identified enzyme was annotated with the ProFunc server (Laskowski et al. 2005). The proteins with known function and proteins with predicted function were manually compiled for the assignment of gene–protein–reaction in the dataset. A draft metabolic network was generated for three individual compartments (CAC-Environment-MAC) by using CellDesigner 5.1 software (Funahashi et al. 2008). The net sum of all production and consumption fluxes was set to zero for each internal metabolite.

### The biochemical formalism of SRCM model

The draft model of SRCM model was updated with metabolic information collected from the MetaCyc Metabolic Pathway Database 22.6 (Caspi et al. 2018) and Kyoto Encyclopedia of Genes and Genomes (KEGG) (Kanehisa et al. 2018). Biochemical reaction formalism was created and optimized using the knowledge on reaction stoichiometry and information on enzyme kinetic data available in the SABIO-reaction kinetics (Wittig et al. 2012) and BRENDA (Schomburg et al. 2013) databases and PUBMED (Additional file 1: Tables S1–S3). Some missing information for the initial concentration of metabolites and enzymes were assigned according to the physiological assumptions of CAC and MAC. Charges of each metabolite in the model were assigned from the metabolic reaction index developed by the Model SEED (Overbeek et al. 2005). All the kinetic parameters in the metabolic model were transformed into a mathematical model by using ordinary differential equations (ODE) (Chou and Voit 2009) and enzyme kinetic functions (Additional file 1). The concentration of metabolites and reaction fluxes were expressed as mmol/min/ml.

### Metabolic flux analysis

The overall reactions with kinetic parameters were merged into a metabolic network by using COPASI 4.11 software (Mendes et al. 2009) and then non-steady-state approximation defined by the stoichiometric matrix using deterministic method (Hoops et al. 2006). The resulted deterministic model captures the collective behaviors of the elements constituting the network, which requires a set of the state variables (Demin and Goryanin 2009). In the deterministic, mass balance equations can be written as,

$$\frac{dx\left( t \right)}{dt} = S.v\left( {x\left( t \right), u\left( t \right),\theta } \right)$$

where x(t) denotes an m-dimensional vector of time-dependent state variables. S is a stoichiometric matrix of dimension m × n. (v(x)t), u(t), (θ) represents an n-dimensional vector of reaction rates, which are dependent on the state variables, a vector of input variables u(t), and a set of parameters θ.

The kinetic rate expressions can be derived from actual reaction mechanisms by approximate expressions capturing the essential quantitative and qualitative features of a reaction. These rate expressions for the transporter and intracellular reaction were described with the generalized mass action and Michaelis–Menten kinetics, respectively. Generalized mass action describes reactions by power law kinetics with non-integer exponents, allowing an analytical steady-state solution to be calculated for linear pathways (Savageau 1971). Michaelis–Menten kinetics can be derived from an on-ordered enzyme mechanism under the assumption of rapid equilibrium between the enzyme and its substrates and products using the following equation (Liebermeister and Klipp 2006a, b; Bajzer and Strehler 2012).

$${\text{V}} = \frac{{{\text{V}}_{ \hbox{max} } {\text{S}}}}{{{\text{Km}} + {\text{S}}}}$$

where v is reaction rate; S is substrate concentration; V_max_ is the maximum rate achieved by the system. Km is substrate concentration at which the reaction rate is half of V_max_. It was used to describe enzyme kinetics where the concentration of the substrate is much higher than the concentration of the enzyme (Chen et al. 2010). Model parameters were determined by minimizing an objective function measuring the difference between experimental data and model predictions (Ljung 1987). Time-scale metabolic simulation data provide important information about the accumulation of metabolites, which implies causal relationships in the metabolic reaction network (Voit 2013). It was performed in a batch culture mode by the Gibson–Bruck Stochastic method (Gibson and Bruck 2000) of the Gillespie algorithm (Gillespie 1976).

A typical objective function to be optimized would be the rate of formation of the desired product. The optimization procedure is a subject to constraints regarding the maximum changes in levels of enzymes and metabolites. A maximal flux value of the objective function was optimized with the ODE. In the metabolic simulation, a reaction involved in methane synthesis was assumed as a maximal objective function whereas reactions involved in glycine–alanine catabolism as minimal objective functions. Every metabolic reaction flux and metabolite concentration in time-series were simulated with the deterministic method and then model robustness examined. The internal steps of the simulation were maximized 10,000 times with an interval size of 1.0.

### Model validation

Model validation is typically done by analyzing the deviation between the measured data and the model outputs (Mendes et al. 2009). The stability and dynamic behavior of this model were calculated by means of eigenvalues, which are originally a special set of scalars associated with a linear system of equations. The degree of change of model properties (model sensitivity) was determined under the curve of state variables in response to a change in the model parameters. Metabolites (xi) and reactions rate (vi) which are catalyzed by enzymes with concentrations *ej*. The elasticity coefficients of this network structure were estimated as below.

$${\text{E}}_{\text{Xj}}^{\text{j}} = \frac{{X_{i} }}{{V_{j} }}.\frac{{\partial V_{j} }}{{\partial X_{j} }}$$

Each elasticity coefficient is a property of an individual enzyme and is therefore independent of the activity of the other enzymes in the pathway. The flux control coefficients of model property, if no single rate-limiting enzyme, were calculated in response to a change in the model parameters.

## Results

### Stickland reactions-coupled methanogenesis

**Fig. 1:**
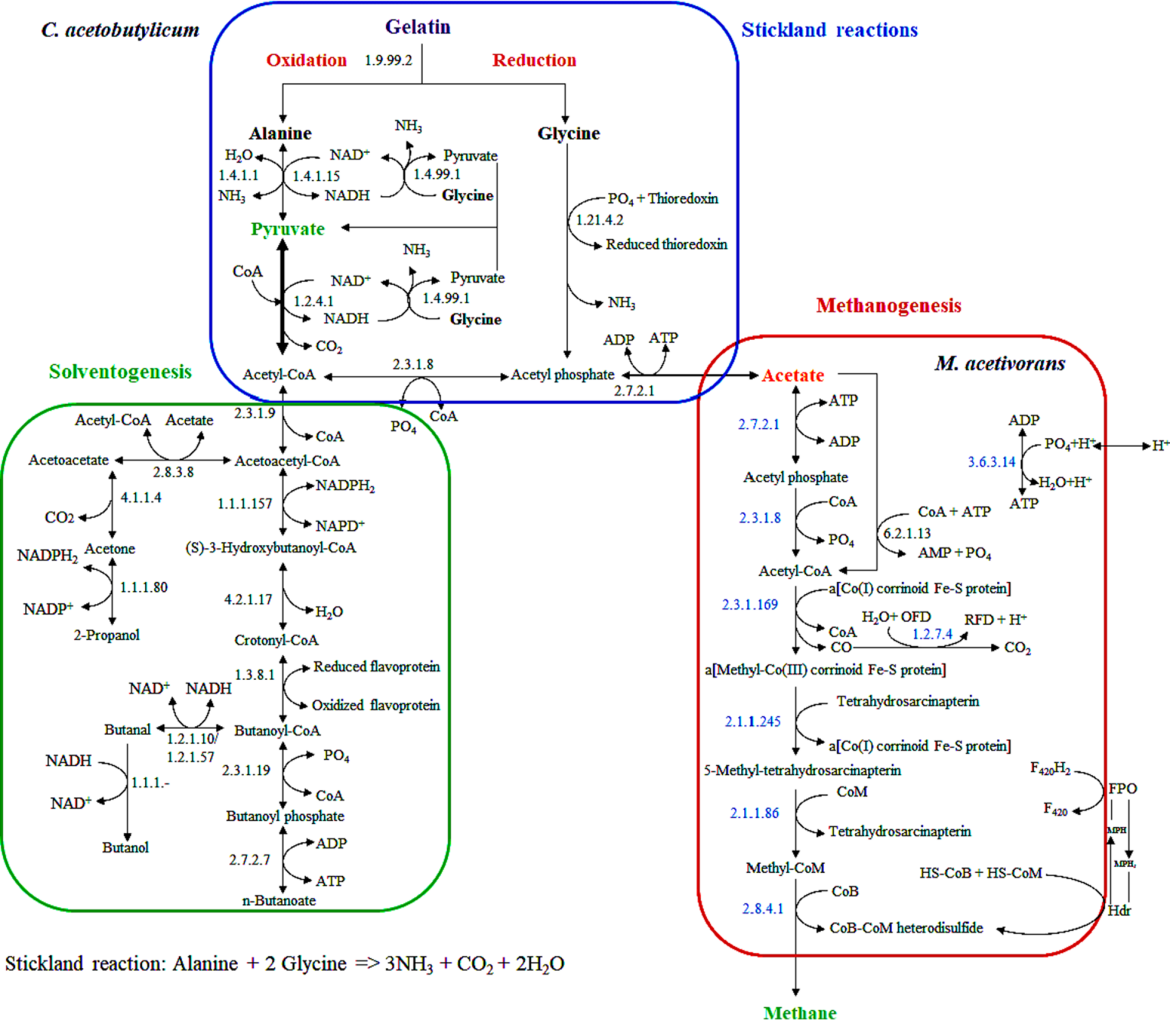
The proposed pathway for the Stickland reactions-coupled methanogenesis in a methanogenic culture

SRCM is used as a metabolic model for our hypothesis testing on the bioconversion of gelatin into methane by a methanogenic culture. Glycine (21.4%), proline (12.4%) and alanine (8.9%) are major amino acids composed in pure gelatin. Extracellular proteases produced by CAC are able to degrade the gelatin and release the small peptides and amino acids to the environment (medium). The liberated amino acids are subsequently catabolized into intracellular metabolites through specialized pathways or the Stickland reactions by CAC. We predicted a catabolic pathway for amino acids showing a connection to acetogenesis and solventogenesis from its genome-scale metabolic model. As shown in Fig. 1, l-alanine from medium is oxidatively deaminated to pyruvate, ammonia, and NADH (reduced NAD^+^) catalyzed by alanine dehydrogenase (EC 1.4.1.1). l-Glycine is deaminated to acetate and ammonia with a reduction of NADH as NAD^+^ by l-amino acid dehydrogenase (EC 1.4.99.1). Reduced thioredoxin and ATP molecule are produced as end-products by glycine reductase and acetate kinase (EC 2.7.2.1), respectively. Acetate is produced from pyruvate via intermediates acetyl-CoA and acetyl-phosphate. This reaction uses two modifiers such as phosphate acetyltransferase (EC 2.3.1.8) and acetate kinase. Pyruvate-formate lyase converts pyruvate to acetyl-CoA by the addition of substrate CoA. Pyruvate dehydrogenase (EC 1.2.4.1) transfers an acetyl group with the addition of electron donor NAD^+^, which reduces to NADH_2_ in the conversion of pyruvate to acetyl-CoA.

Acetate is produced from acetyl-CoA and transported to medium, which is consecutively transformed into methane through methanogenesis. Pyruvate is a main metabolic intermediate formed from amino acid catabolism of CAC. The concentration and reaction flux of it found to determine acetogenesis or solventogenesis in CAC. SRCM is indirectly connected with the synthesis of acetate and *n*-butanoate during acidogenesis whereas production of acetone, propanol, and butanol during solventogenesis (Additional file 1). It simply indicates that there are some metabolic control points for the regulation of acidogenesis, particularly acetate synthesis in favor of methanogenesis in a methanogenic culture or only solventogenesis in CAC.

### Description of membrane transporter

**Fig. 2:**
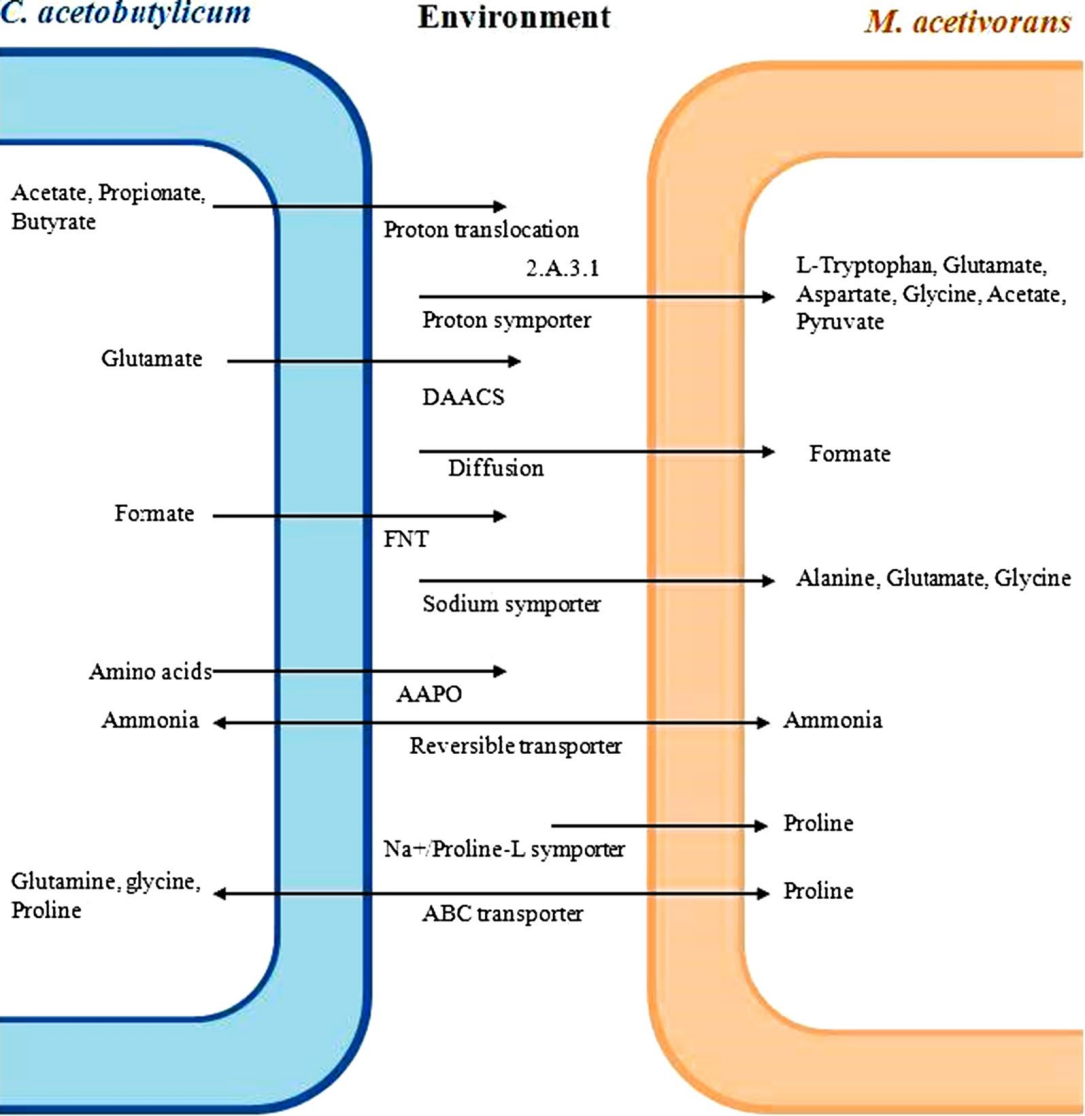
The proposed membrane transporter system for the exchange of substrates and products across the CAC and MAC

Some membrane transporter systems such as diffusion, symporter, proton translocation, anti-bacterial cassette (ABC), formate–nitrite transporter (FNT), amino acid–polyamine-organization (AAPO) and dicarboxylate/amino acid: cation symporter (DAACS) are predicted from the genomes of CAC and MAC. The substrate, as well as product fluxes in related to the SRCM, is mediated by these transporter systems (Fig. 2). Volatile fatty acids except formate are transported from cytoplasm of CAC to the environment via proton translocation and then imported into the cytoplasm of MAC through a proton symporter. AAPO, ABC, DAACS and sodium/proton symporter are common transporter systems for exchange of amino acids through CAC-medium-MAC channel.

### SRCM model properties

Mathematical models have more recently gained great attention for the identification of metabolic engineering targets and associated pathways of industrial microorganisms due to the complexity of microbial metabolism and gene regulation (Almquist et al. 2014; Costa et al. 2016; D’hoe et al. 2018). Reaction kinetics is being the fundamental building block of the kinetic model described by set up of mathematical expressions for the rates of all biochemical species present in the modeled system. The SRCM model developed from this study consists of three compartments in which CAC includes 7 biochemical reactions with 24 metabolites and MAC has 11 intracellular reactions with 37 metabolites. This model contains 6 transport reactions in each compartment. Medium is assumed to be the third compartment in this model. The network structure of the modeled system defines the network of interconnected elements that are assumed to be important for the SRCM. Generally, biomass formalism is a key modeling component for the prediction of growth phenotypes using central metabolism and genome-scale metabolic models.

**Fig. 3:**
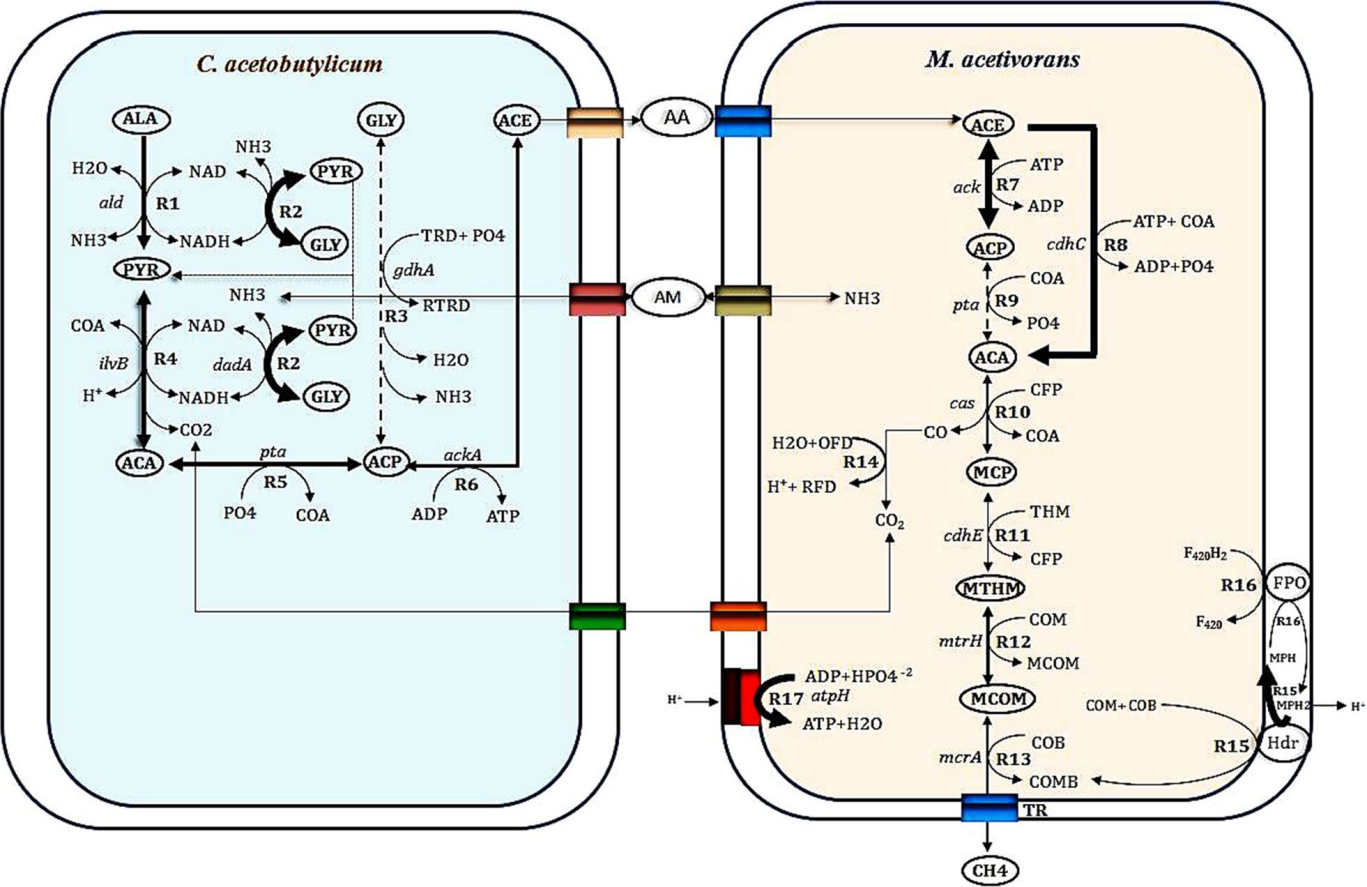
A flux map of the Stickland reactions-coupled methanogenesis under the growth of a methanogenic culture on gelatin as a sole carbon source. Black arrows indicate the feasibility of thermodynamics equilibrium towards the enzymatic reactions and the thickness of the arrow is directionally proportional to the flux rate of each reaction (larger fluxes in the reactions are connected with thicker arrow). A single dotted line indicates zero fluxes of corresponding reactions. The production rate of methane (objective function) depends on the uptake of alanine and glycine (constraints) during the metabolic simulation process. All the reactions in each compartment a different flux value for given experimental parameters (Additional file 1: Tables S2, S3)

Methanogenesis is a chief energetic metabolism in all methanogenic archaea and is also directly associated with the growth rates of them. Despite the prediction of growth rate, we used the SRCM model to compute the methane production rate as a maximized objective function from substrate constraints. As represented in Fig. 3, maximum metabolic fluxes observed in the reactions R2, R4, and R5 of CAC. The rate of these reactions has directed the synthesis of acetate from glycine. We found a drastic flux variability showing to distribute glycine either for pyruvate formation to cellular metabolism or acetate synthesis to maintain the mutualistic interaction between CAC and MAC. We assume that it may be a rate-limiting step for the synthesis of acetate in a methanogenic culture under anaerobic digestion process. In MAC, the maximal reaction rates are predicted for the accumulation of acetyl CoA (R8) and coenzyme M (M15) in the cytoplasm. Acetyl-CoA is a carbon sink for methane production and therefore, the occurrence of reaction flux initiates its accumulation for further energetic metabolism of MAC. Heterodisulfide reductase is an alternative route found in *Methanosarcina* genus for the biosynthesis of coenzyme M in contribution to methane generation. The results of flux distributions explained a steady state approximation requiring for initial degradation of glycine or alanine and glycine–alanine pair through the Stickland reactions.

The ability of SRCM model to incorporate detailed information about reactions gives them a number of advantageous properties. It has predicted the cellular and metabolic behaviors in response to genetic alterations in CAC and MAC. The model’s predictions were shown to compare qualitatively well with previously published experiments. A local parameter sensitivity analysis is to determine the degree of change of some model property in response to a change in the model parameter. It has also opted for identification of suitable targets to rationally design directed metabolic engineering strategies. However, the model’s predictive capability should be verified experimentally by over-expression of metabolic engineering targets, particularly the low flux-control enzymes, for a significant increase in methane production rate. A typical objective function to be optimized would be the rate of formation of methane. The development of competitive cell factories is the expansion of their range of substrates. The present SRCM model contribution aimed for improved utilization of amino acid pairs in CAC. Thus, the SRCM model describes many different levels of metabolic control, regulation, and coordination of biochemical reactions, which are essential for growth-associated methanogenesis of a co-culture of CAC and MAC.

### Time-course simulation for metabolic behavior

**Fig. 4:**
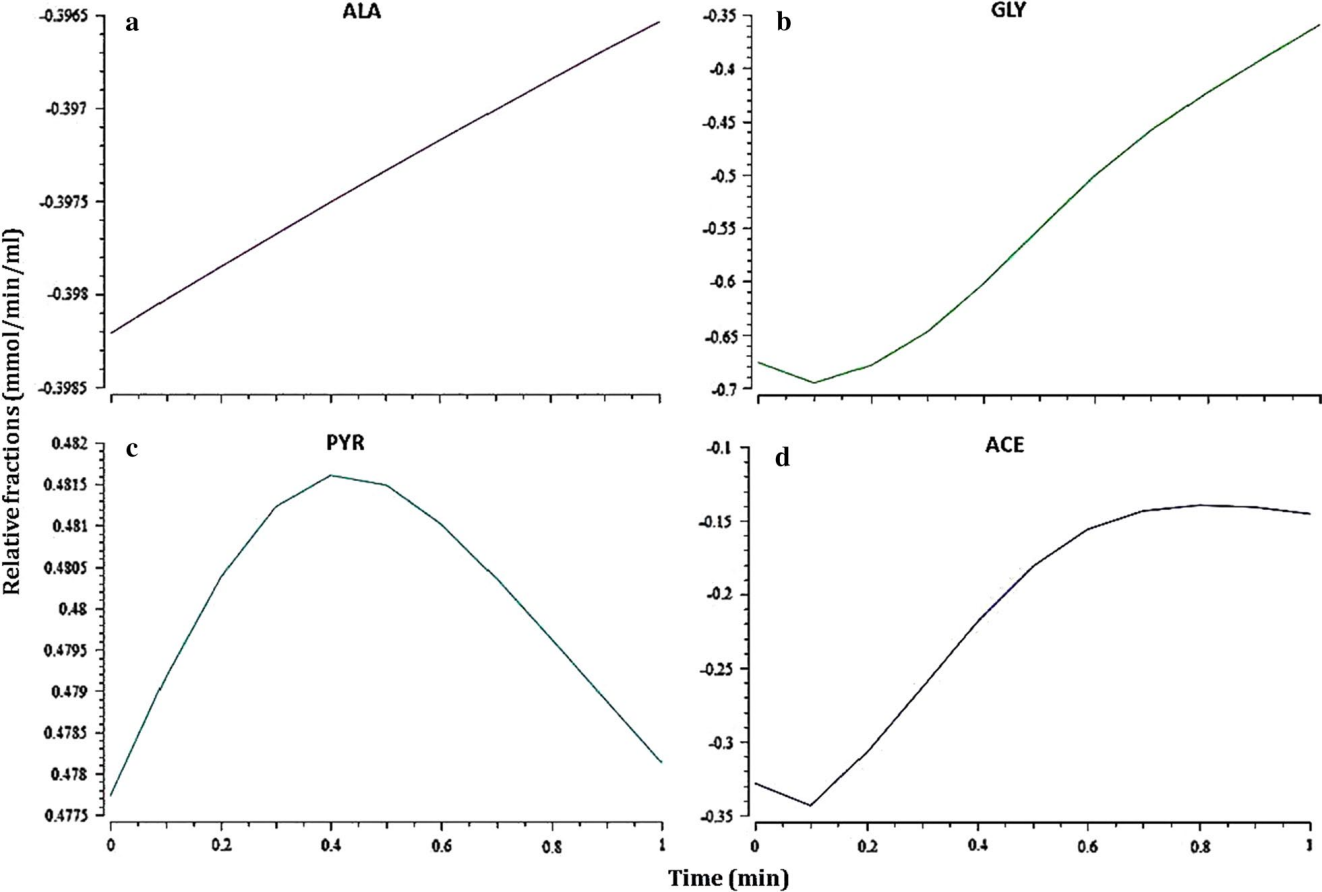
Time-scale simulation of metabolic flux rates in the selected reactions of CAC during the Stickland reaction-coupled methanogenesis. Shown are **a**–**c** uptake rates of alanine, glycine, pyruvate, and acetate production rate (**d**)

**Fig. 5:**
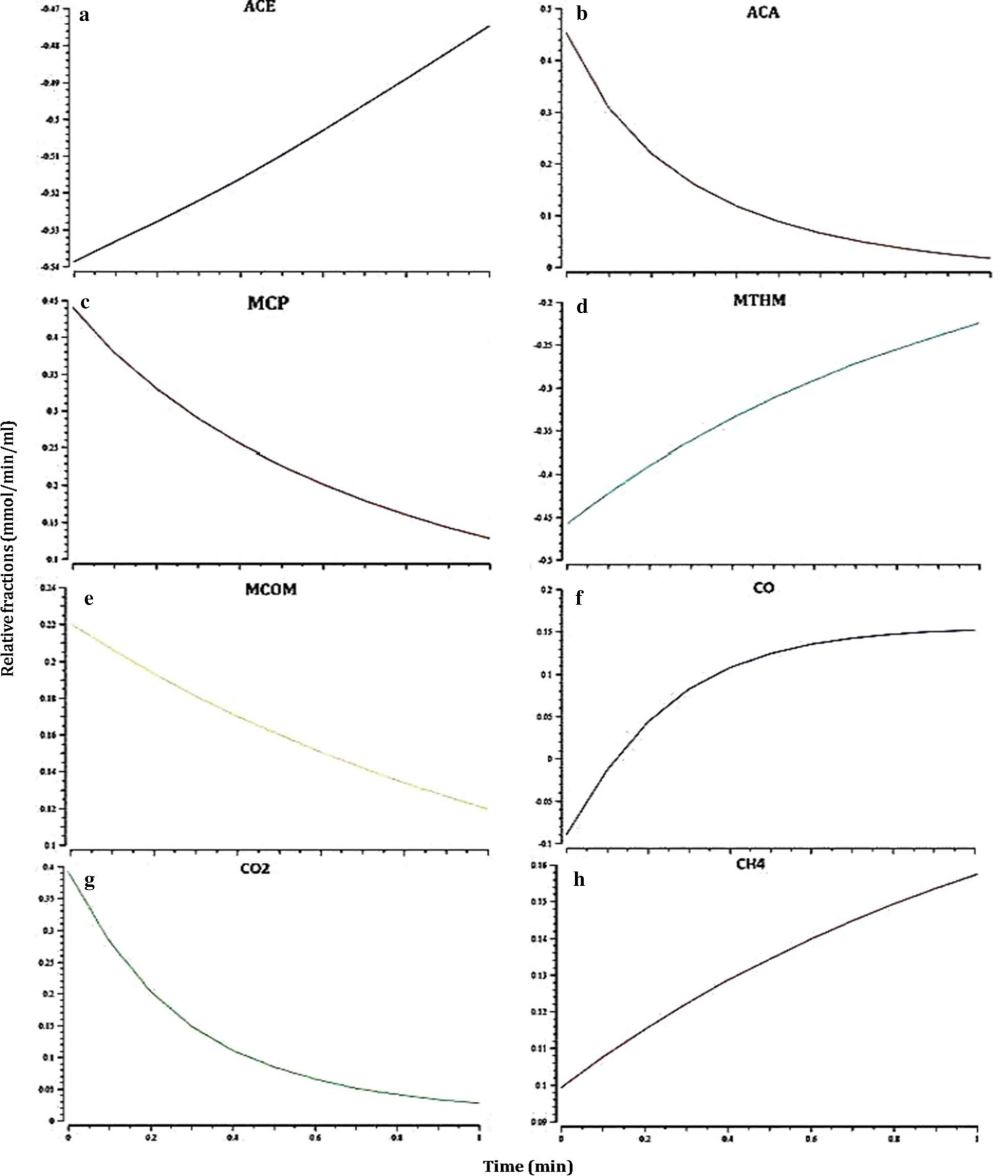
Time-scale simulation of metabolic flux rates in the selected reactions of MAC during the Stickland reaction-coupled methanogenesis. Shown are **a**–**g** uptake rates of acetate, acetyl-CoA, methyl-corrinoid protein, methyl-tetrahydrosarcinapterin, methyl-CoM, CO_2_, CO and methane production rate (**h**)

Many biological processes or systems of importance to biotechnology are non-stationary in their nature, which is characterized by their dependence on time. The rate of reactions that are accountable to maximize the methane production rate was calculated when glycine or alanine and glycine–alanine pair consumed by a methanogenic culture. A gradient flux value has gradually increased the rate of reactions R5 and R6. It may be resulted due to the transport of acetate to the medium. Metabolic flux rate is radically influenced by the decreasing, the rate of reactions R3 (CAC), R9 and R14 (MAC). The depletion of CO_2_ from CO infers the reaction flux of R9. The overall flux distribution has shown to effect on the consumption of glycine alone by glycine reductase-catalyzed reaction and generation of acetyl-CoA for methanogenesis by acetyl-CoA synthetase (Fig. 4). The concentration of alanine and glycine are slowly raised, but not completely assimilated as released from gelatin, indicative of cytoplasmic substrate saturation in CAC. The complete utilization of amino acids from gelatin does not endeavor in the methanogenic culture as a result of low fluxes in the reactions catalyzed by alanine dehydrogenase, amino acid dehydrogenase and glycine reductase (Fig. 5). Pyruvate is almost consumed for acetate synthesis, reflecting that its concentration is directly proportional to the production rate of acetate. The concentration of methane is reached to a maximum when the acetyl-CoA concentration is attained a steady state flux for methanogenesis.

### Robustness of SRCM model for methane production

**Fig. 6:**
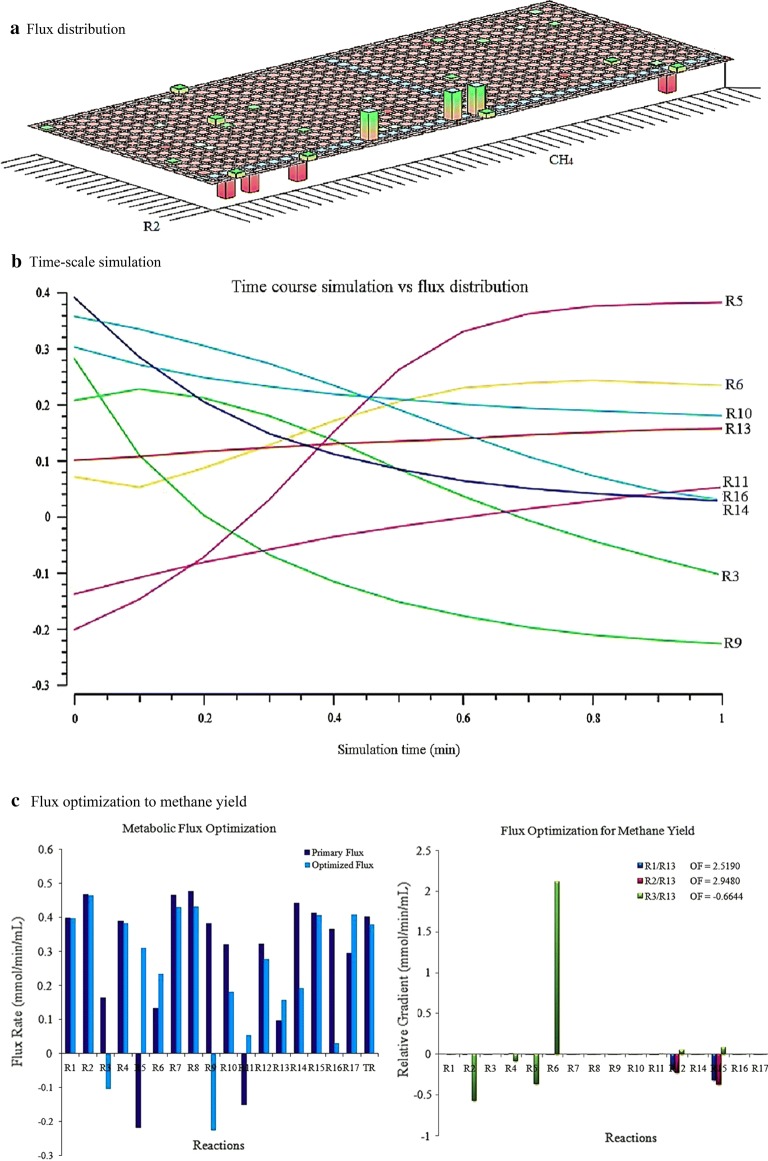
The relative flux distribution of the Stickland reactions-coupled methanogenesis, simulated with kinetic parameters under the growth on gelatin of a methanogenic culture (**a**). A time-scale simulation for calculating an optimized reaction flux rate of the model is shown in **b**. The predicted flux rate of each reaction is proportional to the relative gradients of the respective reactions. The optimized flux rates for the key reactions involving in methanogenesis from amino acids are represented in **c** (left). The production rate of methane (R13) was objectively increased under selective constraints of R1 (alanine) and R2/R3 (glycine), which is shown in **c**. The flux distribution was optimized for a maximal methane rate. Shown are flux values in units of mmol per min per ml. The reaction abbreviations are defined in Additional file 1: Table S1

As shown in Fig. 6a, the primary flux distribution of each reaction is compared with the optimized flux distribution. The primary flux distribution is the overall flux rate of reactions obtained prior to optimization of kinetic parameters and network robustness under pre-steady-state condition. The robustness and consistency of the SRCM model were evaluated for improved methane production rate by simulating it with enzyme kinetic parameters. It shows that metabolic flux rates in the reactions R3 and R9 are decreased as a result of optimizing flux rate of reactions R5 and R11. After flux optimization, we found the production rate of methane increased to 0.1567 mmol methane/min/ml from 0.0967 mmol methane/min/ml (Fig. 6b). This model is more consistent with increased methane production rate (2.5190–2.9480 mmol methane/min/ml) when the alanine–glycine pair is used as a substrate constraint. Nevertheless, it is not reliable to glycine-mediated methanogenesis. It clearly states that this model has good agreement with experimental data for enhanced production of methane from alanine–glycine coupled Stickland reaction.

## Discussion

The protein-based industry typically discharges wastewater containing a huge quantity of proteins and amino acids, which are utilized to produce methane by using defined methanogenic culture (Chellapandi et al. 2008, 2010a; Chellapandi and Uma 2012a, b), a co-culture of *C. collagenovorans* and *M. barkeri* (Jain and Zeikusi 1989) and syntrophic growth of *C. butyricum*, and *M. mazei* under anaerobic conditions (Bizukojc et al. 2010). Acetate and butyrate were common end-products probably arising from the oxidation of amino acids such as alanine or the reduction of glycine in CAC. *Methanosaeta* sp. found to use acetate produced by amino acid degradation and subsequent acidogenesis (Tang et al. 2005). Our study suggested that amino acid catabolism-directed methanogenesis is a central metabolic process of syntrophic methanogenic cultures for the biomethanation of protein-based waste, particularly gelatin.

Glycine reductase is found in the energetically less favorable glycine–serine–pyruvate pathway of *C. acidurici* 9a for glycine degradation (Hartwich et al. 2012). Glycine reductase and pyruvate dehydrogenase widely distributed in many Clostridial species (Arkowitz and Abeles 1991; Bednarski et al. 2001; Graentzdoerffer et al. 2001). Pyruvate–ferredoxin oxidoreductase (Petitdemange et al. 1997) and NADH and NADPH–ferredoxin oxidoreductase (Meinecke et al. 1989) also identified from CAC. The presence of d-proline reductase and the glycine reductase perform the reduction of electron acceptors proline and glycine in *C. difficile*, respectively (Jackson et al. 2006; Bouillaut et al. 2013). We predicted three key enzymes contributing to major functions in the proposed pathway of CAC. *Escherichia coli* amino acid dehydrogenase and Clostridial pyruvate dehydrogenase have shown the functional equivalency to ferredoxin (AAK78284) and pyruvate ferredoxin oxidoreductase (NP_349113), respectively. Anaerobic ribonucleoside triphosphate reductase (YP_004635874), hypothetical protein SMB_G3587 (YP_004638200) and hypothetical protein CA_C1859 (NP_348483) were shown functional analogs to the Clostridial glycine reductase. As shown by our analysis, the proposed amino acid degradation pathway was established in CAC which can be activated through the Stickland reaction.

Stickland reactions are coupled between a reducing and an oxidizing amino acid, and therefore the net production of hydrogen from completely coupled reactions would be zero. The theoretical stoichiometric coefficient for hydrogen is 0.134. This coefficient is a result of 0.174 mol H_2_ produced by oxidation versus 0.040 mol H_2_ consumed by reduction per carbon mole protein consumed. Only about 20% of the hydrogen produced from amino acid fermentation was consumed by the reduction of amino acids (Andreesen et al. 1989). The chosen reaction pathways provided a good prediction of fermentation products. However, these energetically favored reactions should be coupled to hydrogen-consuming methanogens and would not be termed Stickland reactions. Using this model, a co-culture of these organisms has shown to produce methane (2.9480 mmol/min/ml) via alanine–glycine coupled Stickland reaction. About 60% of fermentation reactions were not coupled to amino acid reactions. Consequently, the end-products derived from amino acid catabolism could be consumed by hydrogen-consuming methanogens (Ramasamy and Pullammanmappallil 2001). Our study also predicted a specific pathway actively performing the glutamate and aspartate catabolism in CAC coupled with methanogenesis (Masion et al. 1987) (Additional file 1: Figure S1). Pyruvic acid was a metabolic intermediate that can be catabolized into acetate and *n*-butanoate in the acidogenic phase. Butanoyl-CoA predicted as a metabolic switch for the biosynthesis of *n*-butanoate in accordance with the earlier work (Green et al. 1996). For *n*-propionate biosynthesis, succinate was found as a metabolic switch similar to other Clostridia (Jones and Woods 1986).

Both organisms were shared formate, acetate, pyruvic acid, CO_2_, NH_3_ and H_2_ as common metabolites. The membrane transporter system was fundamental for the exchange of these metabolites to establish the syntrophic growth of both organisms. We predicted several transporter systems including AAPO, ABC, DAACS and sodium/proton symporter to translocate amino acids. Amino acid transport in membrane vesicles of CAC is fused with proteoliposomes containing a functional proton motive force-generating system (Driessen et al. 1989). However, a transporter system of MAC was differed from CAC due to the existence of its unique membrane lipids (Koga et al. 1993). Similarly, a model has been developed to characterize the membrane transport reactions for the excretion of the intermediate metabolite xylitol (Parachin et al. 2011).

Appropriate levels of metabolic enzymes have been optimized for these microbial products using simulation-based study (Gurung et al. 2013; Nigam 2013). Several kinetic models have been used to determine suitable genetic targets for improved production of ethanol (Polisetty et al. 2008), glycerol (Cronwright et al. 2002), lactic acids (Alvarez-Vasquez et al. 2000; Polisetty et al. 2008), antibiotics and amino acids (Lee et al. 2008) in industrial microorganisms. Some kinetic models have been developed for studying improved utilization of substrates including glucose, arabinose, and xylose in bacteria and fungi (Prathumpai et al. 2004; Visser et al. 2004; de Groot et al. 2005; Nishio et al. 2008; Nikolaev 2010). Despite a common methanogenic process, the kinetic model developed from this study provides an insight into the understanding of methane formation from amino acid catabolism in a methanogenic culture.

Results of our study demonstrated how alanine–glycine coupled Stickland reaction is interconnected with methanogenesis for anaerobic digestion of gelatin. Alanine and glycine are reduced by l-amino acid dehydrogenase and glycine reductase, respectively. A typical pathway typical metabolic subsystem is discovered from the CAC genome for aspartate and glutamate catabolism. Pyruvate acts as a metabolic switch to synthesis the acetate, a substrate of methanogenesis and also to predetermine the acidogenesis and solventogenesis. Our study provides the importance and advantage of using the syntrophic degradation of amino acids in the biomethanation process. This model is more reliable for model-driven metabolic engineering of methanogens. It has also consistent with improved methane production from protein-based substrates by a methanogenic culture. The complex biochemistry of cells in a more complete way is represented by kinetic models, where mathematical models should be able to assist in the rational design of cell factory properties or in the production processes. Evaluation of the effects of adding, removing, or modifying molecular components of a cell factory is, therefore, an important one for the design of the bioreactor or fermentation process. Moreover, experimental validation (mass spectral data) will prove our hypothesis on the SRCM of a methanogenic culture for the production of methane biofuel at the large-scale.

## Additional file

**Additional file 1. Fig. S1.** The proposed pathway for single amino acid (aspartate and glutamate) catabolism in C. acetobutylicum. **Table S1.** The reconstructed pathway information of the SRCM model of C. acetobutylicum ATCC874 and M. acetivornas C2A obtained from their genome-scale metabolic models. **Table S2.** Kinetic parameters assigned for substrate and enzymes involved in the reconstructed SRCM model. **Table S3.** Reduced stoichiometry matrix representation for constructed SRCM model.

## Data Availability

The dataset supporting the conclusions of this article is included in the article. All data are fully available without restriction.
