# Supplementary material for: Kinetic modeling of Stickland reactions-coupled methanogenesis for a methanogenic culture
Source: AMB Express. 2019 Jun 10;9:82. doi: 10.1186/s13568-019-0803-8 (PMC6557928; doi:10.1186/s13568-019-0803-8)
Supplement: Supplementary file 1 — Additional file 1. Fig. S1. The proposed pathway for single amino acid (aspartate and glutamate) catabolism in C. acetobutylicum. Table S1. The reconstructed pathway information of the SRCM model of C. acetobutylicum ATCC874 and M. acetivornas C2A obtained from their genome-scale metabolic models. Table S2. Kinetic parameters assigned for substrate and enzymes involved in the reconstructed SRCM model. Table S3. Reduced stoichiometry matrix representation for constructed SRCM model. [file 13568_2019_803_MOESM1_ESM.pdf]

Additional file 1

Kinetic Modeling of Stickland Reactions-Coupled Methanogenesis for a Methanogenic Culture

C. Sangavai[1], M. Bharathi[1], Shilpkar P. Ganesh[2], and P. Chellapandi[1,*]
[1] Molecular Systems Engineering Lab, Department of Bioinformatics, School of Life Sciences, Bharathidasan University, Tiruchirappalli-620 024, Tamil Nadu, India
Tel: +91-431-2407071 Fax:+91-431-2407045 Email: pchellapandi@gmail.com

[2] Department of Microbiology, Biogas Research Centre, Gujarat Vidyapith, Sadra-382 320, Gandhi Nagar, Gujarat, India
*Corresponding author

Description of methanogenesis

At first, acetyl-coA synthetase (EC 2.7.2.1) utilizes CoA and ATP with the release of AMP and phosphate for the conversion of acetate to acetyl-coA. Secondly, ATP-dependent acetate kinase (EC 2.7.2.1) phosphorylates acetate to acetyl phosphate, which is further converted to acetyl-coA by phosphate acetyltransferase (EC 2.3.1.8) with aspiration of CoA to phosphate. Acetyl-coA is shifted to methyl-Co (III) corrinoid Fe-S protein in the presence of Co (I) corrinoid Fe-S protein by acetyl-coA decarbonylase/synthase (EC 2.3.1.169). Acetyl-coA decarbonylase/synthase (EC 2.1.1.245) converts methyl-Co (III) corrinoid Fe-S protein into 5-methyl-tetrahydrosarcinapterin with expel out of Co (I) corrinoid Fe-S protein by utilization of tetrahydrosarcinapterin. A methylgroup from 5-methyl-tertrahydrosarcinapterin is transferred to CoM for the formation of methyl-CoM by tetrahydromethanopterin S-methyltransferase (EC 2.1.1.86). Methyl-CoM reductase (EC 2.8.4.1) is highly specific for coenzyme B that finally converts methyl-CoM to methane.

Acidogenesis and solventogenesis from pyruvate in CAC

Acetyl-coA acetyltransferase (EC 2.3.1.9) converts acetyl-coA to acetoacetyl-coA. Acetoacetyl-coA is converted to acetyl-coA by acetyl-coA transferase (EC 2.8.3.8) and acetoacetate as a product, which further decarboxylated to acetone by acetoacetate decarboxylase (EC 4.1.1.4). Acetone is reduced to 2-proponal by reducing $NADP^+$ to $NADPH_2$. This reaction is catalyzed by $NADP^+$ -dependent oxidoreductase (EC 1.1.1.80). Acetoacetyl-coA is a key intermediate of pyruvate metabolism that initiates the synthesis of n-butanote in a five-step process. Butanoyl-coA is formed from acetoacetyl-coA via intermediates 3-hydroxybutonyl-coA and crotonyl-coA by the consecutive action of 3-hydroxybutyryl-coA dehydrogenase (EC 1.1.1.157), (S)-3-hydroxybutanoyl-coA hydro-lyase (EC 4.2.1.17) and butyryl-coA dehydrogenase (EC 1.3.8.1). Finally, n-butanote is produced from butanoyl-coA via intermediates butanoyl-coA and butanoyl phosphate by two modifying enzymes, phosphate butyryltransferase (EC 2.3.1.19) and butyrate kinase (EC 2.7.2.7). Butanoyl-coA is repeatedly transformed to butanol in two step mechanisms by butanal: NAD^+ oxidoreductase (EC 1.2.1.10) or butanal dehydrogenase (EC 1.2.1.5) with NAD^+ as an acceptor and oxidoreductase (EC 1.1.1.-; R03544) with the reduction of NADH.

Fig. S1 The proposed pathway for single amino acid (aspartate and glutamate) catabolism in C. *acetobutylicum*.

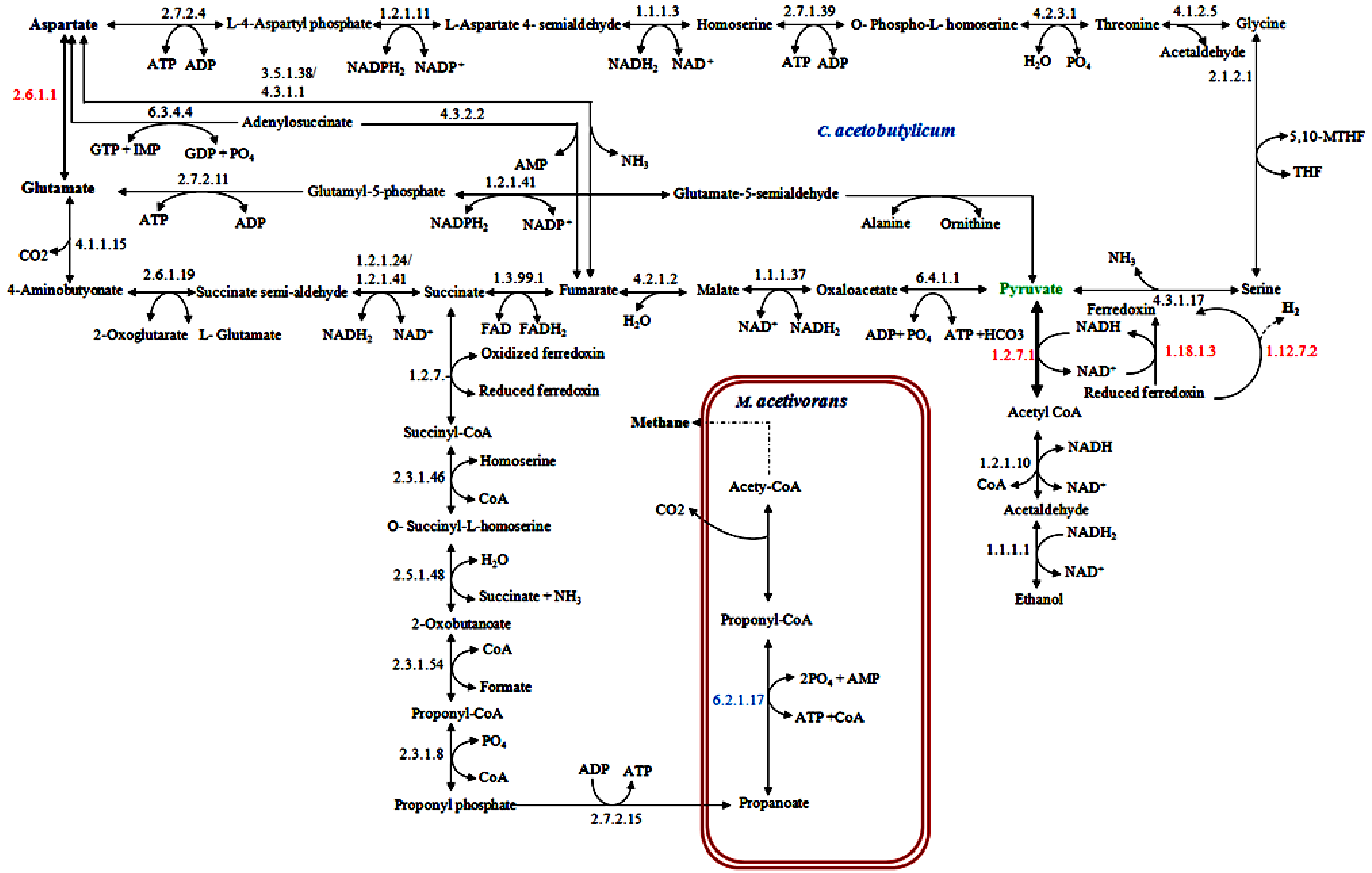

Pathways for single amino acid catabolism in CAC

We have computationally predicted two alternate pathways for acetate synthesis from amino acids, L-aspartate and L-glutamate via pyruvate as depicted in Fig. S3. Pyruvate is resulted from aspartate in four different metabolic directions. Aspartate is directly deaminated into fumarate by aspartate ammonia-lyase (EC 4.3.1.1) in the first direction. Secondly, aspartate transaminase (EC 2.6.1.1) performs the transamination reaction to convert the aspartate to glutamate. Thirdly, fumarate is synthesized from aspartate with the intermediate of adenylosuccinate by adenylosuccinate synthase (EC 6.3.4.4) and adenylosuccinate lyase (EC 4.3.2.2). Lastly, aspartate is phosphorylated to L-4-aspartyl phosphate by aspartate kinase (EC 2.7.2.4) through ATP phosphorylation. L-4-Aspartyl phosphate is converted into L-aspartate 4-semialdehyde by aspartate-semialdehyde dehydrogenase (EC 1.2.1.11) with the reduction of $NADPH_2$ to $NADP^+$. Homoserine dehydrogenase (EC 1.1.1.3) converts L-aspartate 4- semialdehyde into homoserine with the reduction of $NADH_2$ to NAD^+. Homoserine is phosphorylated into O-phospho-L- homoserine by homoserine kinase (EC 2.7.1.39). Threonine synthase (EC 4.2.3.1) acts on O- phospho-L-homoserine to synthesize the L-threonine by the addition of water with the removal of phosphate. L-Threonine is aldolized into glycine and acetaldehyde as a by-product by L-threonine aldolase (EC 4.1.2.5). Glycine is switched to serine by serine-hydroxymethyltransferase (EC 2.1.2.1) to shift methyl group by reducing 5, 10-methyl tetrahydrofolate to tetrahydrofolate. Finally, serine is dehydrated to pyruvate by L-serine dehydratase (4.3.1.17).

Glutamate is catabolized to pyruvate and switched to propanoate via succinate. Glutamate-5-kinase (EC 2.7.2.11) catalyzes the ATP-dependent conversion of glutamate to glutamyl-5-phosphate. Glutamate-5-semialdehyde dehydrogenase (EC 1.2.1.41) produces glutamate-5-semialdehyde from glutamyl-5-phosphate with oxidation of $NADPH_2$ as $NADP^+$. Subsequently, pyruvate is produced by coordinate with alanine, which is then converted to ornithine. Glutamate is decarboxylated to 4-aminobutyonate and formed succinate semialdehyde by the consecutive actions of glutamate decarboxylase (EC 2.6.1.19) and succinate-semialdehyde dehydrogenase (EC 1.2.1.24).Glutamate-5-semialdehyde dehydrogenase (EC 1.2.1.41) catalyzes the oxidation of succinate semialdehyde to succinate. Fumarate is formed from succinate by succinate dehydrogenase (EC 1.3.99.1), which dehydrated to malate with the catalytic action of fumarate hydratase (EC 4.2.1.2). Malate dehydrogenase (EC 1.1.1.37) carried out the conversion of malate to oxaloacetate by reducing NAD^+ to $NADH_{2.}$ Pyruvate carboxylase (EC 6.4.1.1) channeled the conversion with substrate level phosporylation of oxaloacetate to pyruvate, which followed by synthesis of acetyl-coA through pyruvate-ferredoxin oxidoreductase (EC 1.2.7.1). Pyruvate is oxidatively decarboxylated to acetyl co-A by pyruvate-ferredoxin oxidoreductase (EC 1.2.7.1). In the meanwhile, the reduced ferredoxin binds with NAD^+ and routing out for the production of H_2 by ferredoxin-NAD^+ reductase (EC 1.18.1.3 and EC 1.12.7.2). We have shown that succinate acts as a metabolic switch to produce propanoate. Succinate is shifted to succinyl co-A by reduction of ferredoxin. Succinyl co-A is converted to succinyl-L-homoserine catalyzed by homoserine O-succinyltransferase (EC 2.3.1.46). 2-Oxobutanoate is synthesized from succinyl-L-homoserine by cystathionine gamma-synthase (EC 2.5.1.48) with release of succinate and ammonia. Using co-A, pyruvate-formate lyase (EC 2.3.1.54) acts on 2-oxobutanoate to convert to proponyl co-A, which further phosphorylated to proponyl

phosphate by phosphotransacetylase (EC 2.3.1.8). Propanoate is an end product of CAC derived from proponyl phosphate that would serve as one of the methanogenic substrates for MAC after converting to proponyl Co-A.

Table S1: The reconstructed pathway information of the SRCM model of *C. acetobutylicum* ATCC874 and *M. acetivornas* C2A obtained from their genome-scale metabolic models

Locus tag	Protein	Gene	Abbn.	Function	Reaction	RXN. ID
***Clostridium acetobutylicum* ATCC874**						
CLOST_0512|CLOST_0519	L-alanine dehydrogenase	*Ald*	E1	1.4.1.1	L-Alanine + H_2O + NAD^+ = Pyruvate + NH_3 + NADH + H^+	R1
CLOST_2391	Pyruvate-formate lyase	*pflB*		2.3.1.54	Acetyl-CoA + Formate = Acetyl-CoA + Pyruvate	R2
CLOST_0110|CLOST_0112|CLOST_0113|CLOST_0115|CLOST_0116	Glycine reductase	*gdhA*	E3	1.21.4.2	L-Glycine + Phosphate + Thioredoxin <=> Acetyl phosphate + NH_3 + Reduced Thioredoxin + H_2O	R3
CLOST_1166	pyruvate dehydrogenase ($NADP^+$)	*acoL*	E4	1.2.1.51	Pyruvate + CoA + $NADP^+$ <=> Acetyl-CoA + CO_2 + NADPH	R4
CLOST_1915	Phosphate acetyltransferase	*pta*	E5	2.3.1.8	Acetyl-CoA + Phosphate <=> CoA + Acetyl phosphate	R5
CLOST_1354|CLOST_1029	Acetate kinase	*ackA*	E6	2.7.2.1	ADP + Acetyl phosphate <=> ATP + Acetate	R6
Methanosarcina acetivornas C2A						
MA3606	Acetate kinase	*ack*	E7	2.7.2.1	ATP + Acetate <=> ADP + Acetyl phosphate	R7
MA1014/MA1015/MA1016/MA3860/MA3861/MA3862	Acetyl-CoA synthetase	*cdhC / cdhB / cdhA*	E8	6.2.1.13	ATP + Acetate + CoA <=> ADP + Phosphate + Acetyl-CoA	R8

MA3607	Phosphate acetyltransferase	*pta*	E9	2.3.1.8	Acetyl phosphate + CoA <=> Acetyl-CoA + Phosphate	R9
MA0859/MA4384	CO-methylating acetyl-CoA synthase	*cas*	E10	2.3.1.169	Acetyl-CoA + Corrinoid protein <=> CoA + CO + Methylcorrinoid protein	R10
MA4558/MA1494	Methyltransferase	*cdhE/cdhD*	E12	2.1.1.245	a [methyl-Co(III) corrinoid Fe-S protein] + Tetrahydrosarcinapterin <=> a [Co(I) corrinoid Fe-S protein] + 5-Methyltetrahydrosarcinapterin	R11
MA0269/MA0272/MA0276/MA0274/ MA0270/ MA0273	Tetrahydromethanopterin S-methyltransferase	*mtrH*	E13	2.1.1.86	5-Methyl-tetrahydrosarcinapterin + coenzyme M <=> Methyl-CoM + Tetrahydrosarcinapterin	R12
MA4546/MA4547/ MA4550	Methyl-CoM reductase	*mcrA/mcrB/mcrG*	E14	2.8.4.1	Methyl-CoM + CoB <=> CoM-S-S-CoB + Methane	R13
MA1309/MA3282	CO dehydrogenase	*cooS*	E11	1.2.7.4	CO + H_2O + Ferredoxin => CO_2 + H^+ + Reduced Ferredoxin	R14
MA0687/MA0688	Heterodisulfide reducatse	*hdrDE*	E16	1.8.98.1	CoMB + MPH_2 <=> CoM + CoB + MP + H^+	R15
MA1495/ MA1496/ MA1497/ MA1498/ MA1499/ MA1500/ MA1503/ MA1504/ MA1505/ MA1506/ MA1507	$F_{420}H_2$ dehydrogenase	*fpoA/ fpoB/ fpoC/ fpoD/ fpoH/ fpoI/ fpoK/ fpoL fpoM/fpoN/fpoO*	E15		$F_{420}H_2$ + MP <=> F_{420} + MPH_2 + H^+	R16
MA2433/	ATP synthase	*atpH/*	E17	3.6.3.14	ADP + Phosphate + H^{+out} <=> ATP + H2O + H^{+in}	R17

MA2434<br>/MA2435<br>/MA2436<br>/MA2437/<br>MA2440/<br>MA2441/<br>MA4152/M<br>A4153/MA<br>4154/<br>MA4155/<br>MA4156/<br>MA4157/<br>MA4158/<br>MA4159/<br>MA4160		*atpI/*<br>*atpC/*<br>*atpE/*<br>*atpC/*<br>*atpF/*<br>*atpA/*<br>*atpB/*<br>*atpD*				
**Transporter**						
CH_4	Auto transporter	CH_4{MAC} <=> CH_4{MED}; TME				TR

Table S2: Kinetic parameters assigned for substrate and enzymes involved in the reconstructed SRCM model

**EC**	**Metabolite**	***Km* (mM)**	***Vmax* (mM)**	**Initial conc. (mM)**	**Specific activity (U/mg of protein)**	**Organisms**	**References**
1.4.99.1	Glycine	5.8	0.5	0.58	10	*Clostridium acetobutylicum*	Based on the physiological hypothesis and compared with other enzyme kinetic data
	Pyruvate	1.7	0.5	0.17			
	NADH	0.1	0.1	2			
	Ammonia	28.2	0.5	2.82			
	NAD^+	0.23	0.1	0.2			
1.21.4.2	Glycine	5.8	0.5	1.3	349	*Eubacterium acidaminophilum*	Meyer et al. 1995
	PO_4	2.8	0.5	0.58			
	Thioredoxin	1.69	0.1	0.169		*Clostridium pasteurianum*	Hammel et al. 1983
	Reduced Thioredoxin	0.08	0.1	0.008		*Clostridium pasteurianum*	Hammel et al. 1983
	Ammonia	5	0.5	2.82		*Clostridium acetobutylicum*	Senger and Papoutsakis 2008
	Acetyl phosphate	0.032	0.5	0.0024			
1.4.1.1	L-alanine	18.9	0.188	1.89	0.049	*Bacillus subtilis, Clostridium acetobutylicum*	Ohashima and Soda 1979; Senger and Papoutsakis 2008; Meinecke et al. 1989
	NAD^+	0.23	0.1	0.2			
	NADH	0.01	0.1	2			
	Ammonia	28.2	0.5	2.82			
1.2.4.1	Pyruvate	1.7	0.91	0.17	19.5		
	CoA	0.037	0.1	0.2			
	NAD_+	1	0.1	0.2		*Clostridium kluyveri*	Wang et al. 2010
	NADH	0.025	0.1	2			
	CO_2	10	0.5	1			
2.3.1.8 (CAC)	Acetyl-CoA	0.023	0.5	3	570		
	PO_4	2.8	0.5	0.28		*Clostridium acetobutylicum*	Senger and Papoutsakis 2008

	CoA	0.03	0.1	0.2		*Eubacterium Thermotoga maritima*	Bock and Glasemacher 1999
2.3.1.8 (MAC)	Acetyl phosphate	89	0.5	8.9	9006	*Methanosarcina thermophila*	Latimer and Ferry 1993; Rasche et al. 1997; Lawrence and Ferry 2006
	CoA	0.034	0.1	0.15			
	PO_4	0.742	0.5	0.28			
2.7.2.1(C AC)	Acetyl phosphate	0.024	0.5	0.0024	27.7	*Eubacterium Thermotoga maritime, Clostridium acetobutylicum*	Bock and Glasemacher 1999; Diez-Gonzalez et al. 1996
	ADP	3	0.1	0.3		*Eubacterium Thermotoga maritime, Methanosarcina thermophila*	Bock and Glasemacher 1999; Jablonski and Ferry 1991
	ATP	0.16	0.1	0.016		*Eubacterium Thermotoga maritima*	Bock and Glasemacher 1999
	CoA	1.5	0.1	0.2		*Eubacterium Thermotoga maritima*	Bock and Glasemacher 1999
2.7.2.1 (MAC)	Acetate	40	0.5	4	12	*Eubacterium Thermotoga maritima*	Bock and Glasemacher 1999
	ATP	2.8	0.1	0.3		*Methanosarcina thermophila*	Jablonski and Ferry 1991
	ADP	1	0.1	0.1			
6.2.1.13	ATP	0.133	0.1	0.3	75	*Archaeoglobus fulgidus*	Musfeldt and Schönheit 2002
	Acetate	0.00258	0.5	4			
	CoA	0.027	0.1	0.15			
2.3.1.169	Acetyl-coA	0.28	0.5	0.028	0.12	*Clostridium thermoaceticum Carboxydothermus hydrogenoformans*	Roberts et al. 1992 Svetlitchnyi and Dobbek (2004)
	A[Co(I)corrinoid Fe-S protein]	0.1	0.5	1			
	CoA	4.3	0.1	0.15		*Moorella thermoacetica*	Lu et al. 1990
1.2.7.4	CO	0.053	0.5	0	133.6	*Oligotropha carboxidovorans, Methanosarcina barkeri*	Meyer and Schlegel 1980 Allen et al. 1999;

							Krzycki and Zeikus 1984; Grahame and Stadtman 1987
	H_2O	1	0.1	0.1			
	Oxidized ferredoxin	0.5	0.1	0.1		*Clostridium kluyveri*	Wang et al. 2010
	CO_2	40	0.5	1			
	Reduced ferredoxin	0.01	0.1	1			
	H^+	20	0.1	2.0			Baumer et al. 2000
2.1.1.245	A[Methyl Co(III)corrinoid Fe-S protein]	0.012	0.5	0.1	0.9	*Moorella thermoacetica*	Seravalli et al. 1999
	Tetrahydrosarcin apterin	0.002	0.5	0.37			
	A[Co(I)corrinoid Fe-S protein]	0.002	0.5	1			
2.1.1.86	5-Methyl-tetrahydrosarcina pterin	0.00085	0.5	1	1	*Methanobacterium thermoautotrophicum*	Allen et al. 1999; Gunsalus and Wolfe 1980
	CoM	1	0.1	0.1			
	Tetrahydrosarcin apterin	3.7	0.5	0.37			
2.8.4.1	Methyl-CoM	0.6	0.5	0.1	0.00046		
	CoB	0.1	0.1	0.01			
	CoB-CoM heterodisulfide	1.8	0.5	0.18		*Methanosarcina mazei*	Abken et al. 1998
	Methane	0.066	0.34	1			
1.8.98.1	CoB-CoM heterodisulfide	0.1	0.01	0.18	0.62	*Methanobacterium thermoautotrophicum*	Hedderich et al. 1990
	MPH_2	0.092	0.009 2		1.2	*Methanosarcina barkeri*	Heiden et al. 1994

	CoM	0.8	0.08	0.1			
	CoB	0.25	0.025	0.01			
	H_2	0.03	0.003				
F420H2 dehydrogenase	$F_{420}H_2$	0.0020	0.5	0.2	1.4		Baumer et al. 2000
	MP	0.25	0.5	0.025		*Methanosarcina mazei*	Abken et al. 1998
	F_{420}	0.043	0.1	1		*Methanosarcina barkeri*	Brömmelstroet et al. 1990
	MPH_2	0.28	0.5	1.2			
	H^+	20	0.1	2.0			Baumer et al. 2000
3.6.3.14	ADP	0.025	0.1	0.1	2.7	*Acetobacterium woodii, Escherichia coli*	Reidlinger and Muller 1994; Lino et al. 2009
	PO_4	3.2	0.5	0.28			
	ATP	0.052	0.1	0.3			

Table S3 Reduced stoichiometry matrix representation for constructed SRCM model

	H+	ACP	NAD+	MACA	H2O	MACE	MCOM	MP	NH3	CFP	MADP	MACA	CO	MTHM	MACE	COB	PO4
R1	0	0	-1	0	-1	0	0	0	1	0	0	0	0	0	0	0	0
R2	0	0	1	0	-1	0	0	0	1	0	0	0	0	0	0	0	0
R3	0	1	0	0	1	0	0	0	1	0	0	0	0	0	0	0	-1
R4	0	0	-1	0	0	0	0	0	0	0	0	1	0	0	0	0	0
R5	0	1	0	0	0	0	0	0	0	0	0	-1	0	0	1	0	-1
R6	0	-1	0	0	0	-1	0	0	0	0	0	0	0	0	0	0	0
R7	0	0	0	0	0	-1	0	0	0	0	1	0	0	0	0	0	0
R8	0	0	0	1	1	0	0	0	0	0	1	0	0	0	0	0	0
R9	0	0	0	1	1	0	0	0	0	0	0	0	0	0	0	0	0
R10	0	0	0	1	-1	0	0	0	0	1	0	0	1	0	0	0	0
R11	0	0	0	0	0	0	0	0	0	1	0	0	0	0	0	0	0
R12	0	0	0	0	0	0	1	0	0	0	0	0	0	0	0	1	0
R13	0	0	0	0	0	0	-1	0	0	0	0	0	-1	0	0	0	0
R14	1	0	0	0	0	0	0	0	0	0	0	0	0	0	0	-1	0
R15	1	0	0	0	0	0	0	1	0	0	0	0	0	0	0	0	0
R16	1	0	0	0	0	0	0	-1	0	0	-1	0	0	0	0	0	0
R17	-1	0	0	0	0	0	0	0	0	0	0	0	0	0	-1	0	0
TR	0	0	0	0	0	1	0	0	0	0	0	0	0	0	0	0	0

References

1. Abken, H., Tietze, M., Brodersen, .J., Bäumer, S., Beifuss, U., Deppenmeier, U. (1998). *Journal of Bacteriology*, 180, 2027-2032.
2. Allen, J.R., Clark, D.D., Krum, J.G., Ensign, S.A. (1999). PNAS, 96, 8432-8437.
3. Baumer, S., Ide, T., Jacobi, C., Johann, A., Gottschalk, G., Deppenmeier, U. (2000). *The Journal of Biological Chemistry*, 275, 17968-73.
4. Bock, A., Glasemacher, J., Schmidt, R., Schönheit, P. (1989). *Journal of Bacteriology*, 181, 1861-1867.
5. Brömmelstroet, B., Hensgens, C.M., Geerts, W., Keltjens, J.T., van der Drift, C., Vogels, G.D. (1990). *Journal of Bacteriology*, 172(2), 564–571.
6. Diez-Gonzalez, F., Russell, J.B., Hunter, J.B. (1996). *Archives of Microbiology*, 166(6), 418-20.
7. Grahame, D.A., Stadtman, T.C. (1987). *The Journal of Biological Chemistry*, 262, 3706-12.
8. Gunsalus, R.P., Wolfe, R.S. (1980). *The Journal of Biological Chemistry*, 255, 1891-5.
9. Hammel, K.E., Cornwell, K.L., Buchanant, B.B. (1983). *Biochemistry*, 80, 3681-3685.
10. Hedderich, R., Berkessel, A., Thauer, R.K. (1990). *European Journal of Biochemistry*, 193, 255-61.
11. Heiden, S., Hedderich, R., Setzke, E., Thauer, R.K. (1994). *European Journal of Biochemistry*, 221, 855-861.
12. Jablonski, P.E., Ferry, J.E. (1991). *Journal of Bacteriology*, 173(8), 2481-7.
13. Krzycki, J.A., Zeikus, J.G. (1984). *Journal of Bacteriology*, 231-237.
14. Latimer, M.T., Ferry, J.G. (1993). *Journal of Bacteriology*, 175(21), 6822-9.
15. Lawrence, S.H., Ferry, J.G. (2006). *Journal of Bacteriology*, 188(3), 1155-8.
16. Lu, W.P., Harder, S.R., Ragsdale, S.W. (1990). *The Journal of Biological Chemistry*, 265(6), 3124-33.
17. Meinecke, B., Bertram, J., Gottschalk, G. (1989). *Archives of Microbiology*, 152(3), 244-250.
18. Meyer, M., Granderath K., Andreesen J.R. (1995). *European Journal of Biochemistry*, 234, 184-191.
19. Meyer, O., Schlegel, H.G. (1980). *Journal of Bacteriology*, 141(1), 74-80.
20. Musfeldt, M., Schönheit, P. (2002). *Journal of Bacteriology*, 184(3), 636-44.
21. Ohashima, T., Soda, K. (1997). *European Journal of Biochemistry*, 100, 29-39.
22. Rasche, M.E., Smith, K.S., Ferry, J.G. (1997). *Journal of Bacteriology*, 179, 7712-7.
23. Reidlinger, .J., Mullerf, V. (1994). *European Journal of Biochemistry*, 223, 275-283.
24. Roberts, J.R., Lu, W.P., Ragsdale, S.W. (1992). *Journal of Bacteriology*, 174(14), 4667-76.
25. Senger, R.S., Papoutsakis, E.T. (2008). *Biotechnology and Bioengineering*, 101.
26. Svetlitchnyi, V., Dobbek, H., Meyer-Klaucke, W., Meins, T., Thiele, B., Römer, P., Huber, R., Meyer, O. (2003). Proc Natl Acad Sci U S A. 101(2), 446-51.
27. Wang, S., Huang, H., Moll, J., Thauer, R.K.(2010). *Journal of Bacteriology*, 192, 5115-5123.

Ordinary differential equations for simulation of SRCM model in this study

$$\frac{d([\text{ACA\{CAC\}}]\cdot V_{CAC})}{dt} = +V_{CAC}\cdot\left(\frac{\frac{0.5\cdot[\text{PYR}]}{1.7}+\frac{0.5\cdot[\text{COA\{CAC\}}]}{0.037}+\frac{0.5\cdot[\text{"NAD+"}]}{1}-\frac{0.4\cdot[\text{ACA\{CAC\}}]}{2}+\frac{0.4\cdot[\text{CO2\{CAC\}}]}{10}+\frac{0.4\cdot[\text{NADH}]}{0.025}+\frac{0.4\cdot[\text{"H+\{CAC\}"}]}{10}}{1+\frac{[\text{PYR}]}{1.7}+\frac{[\text{COA\{CAC\}}]}{0.037}+\frac{[\text{"NAD+"}]}{1}+\frac{[\text{ACA\{CAC\}}]}{2}+\frac{[\text{CO2\{CAC\}}]}{10}+\frac{[\text{NADH}]}{0.025}+\frac{[\text{"H+\{CAC\}"}]}{10}}\right)$$
$$-V_{CAC}\cdot\left(\frac{\frac{0.5\cdot[\text{ACA\{CAC\}}]}{2}+\frac{0.5\cdot[\text{PO4\{CAC\}}]}{2.8}-\frac{0.4\cdot[\text{COA\{CAC\}}]}{0.03}+\frac{0.4\cdot[\text{ACP\{CAC\}}]}{89}}{1+\frac{[\text{ACA\{CAC\}}]}{2}+\frac{[\text{PO4\{CAC\}}]}{2.8}+\frac{[\text{COA\{CAC\}}]}{0.03}+\frac{[\text{ACP\{CAC\}}]}{89}}\right)$$

$$\frac{d([\text{ACE\{CAC\}}]\cdot V_{CAC})}{dt} = -([\text{ACE\{CAC\}}]\cdot 0.1)$$
$$+V_{CAC}\cdot\left(\frac{\frac{0.5\cdot[\text{ADP\{CAC\}}]}{3}+\frac{0.5\cdot[\text{ACP\{CAC\}}]}{0.024}-\frac{0.4\cdot[\text{ATP\{CAC\}}]}{0.16}+\frac{0.4\cdot[\text{ACE\{CAC\}}]}{40}}{1+\frac{[\text{ADP\{CAC\}}]}{3}+\frac{[\text{ACP\{CAC\}}]}{0.024}+\frac{[\text{ATP\{CAC\}}]}{0.16}+\frac{[\text{ACE\{CAC\}}]}{40}}\right)$$

$$\frac{d([\text{ACP\{CAC\}}]\cdot V_{CAC})}{dt} = +V_{CAC}\cdot\left(\frac{\frac{0.5\cdot[\text{GLY}]}{5.8}+\frac{0.5\cdot[\text{PO4\{CAC\}}]}{2.8}+\frac{0.5\cdot[\text{TRD}]}{1.69}-\frac{0.4\cdot[\text{ACP\{CAC\}}]}{0.032}+\frac{0.4\cdot[\text{NH3}]}{5}+\frac{0.4\cdot[\text{RTRD}]}{0.08}+\frac{0.4\cdot[\text{H2O\{CAC\}}]}{1}}{1+\frac{[\text{GLY}]}{5.8}+\frac{[\text{H2O\{CAC\}}]}{1}+\frac{[\text{PO4\{CAC\}}]}{2.8}+\frac{[\text{TRD}]}{1.69}+\frac{[\text{ACP\{CAC\}}]}{0.032}+\frac{[\text{NH3}]}{5}+\frac{[\text{RTRD}]}{0.08}}\right)$$
$$+V_{CAC}\cdot\left(\frac{\frac{0.5\cdot[\text{ACA\{CAC\}}]}{2}+\frac{0.5\cdot[\text{PO4\{CAC\}}]}{2.8}-\frac{0.4\cdot[\text{COA\{CAC\}}]}{0.03}+\frac{0.4\cdot[\text{ACP\{CAC\}}]}{89}}{1+\frac{[\text{ACA\{CAC\}}]}{2}+\frac{[\text{PO4\{CAC\}}]}{2.8}+\frac{[\text{COA\{CAC\}}]}{0.03}+\frac{[\text{ACP\{CAC\}}]}{89}}\right)$$
$$-V_{CAC}\cdot\left(\frac{\frac{0.5\cdot[\text{ADP\{CAC\}}]}{3}+\frac{0.5\cdot[\text{ACP\{CAC\}}]}{0.024}-\frac{0.4\cdot[\text{ATP\{CAC\}}]}{0.16}+\frac{0.4\cdot[\text{ACE\{CAC\}}]}{40}}{1+\frac{[\text{ADP\{CAC\}}]}{3}+\frac{[\text{ACP\{CAC\}}]}{0.024}+\frac{[\text{ATP\{CAC\}}]}{0.16}+\frac{[\text{ACE\{CAC\}}]}{40}}\right)$$

$$\frac{d([\text{ADP\{CAC\}}]\cdot V_{CAC})}{dt} = -V_{CAC}\cdot\left(\frac{\frac{0.5\cdot[\text{ADP\{CAC\}}]}{3}+\frac{0.5\cdot[\text{ACP\{CAC\}}]}{0.024}-\frac{0.4\cdot[\text{ATP\{CAC\}}]}{0.16}+\frac{0.4\cdot[\text{ACE\{CAC\}}]}{40}}{1+\frac{[\text{ADP\{CAC\}}]}{3}+\frac{[\text{ACP\{CAC\}}]}{0.024}+\frac{[\text{ATP\{CAC\}}]}{0.16}+\frac{[\text{ACE\{CAC\}}]}{40}}\right)$$

$$\frac{d([\text{ALA}]\cdot V_{CAC})}{dt} = -V_{CAC}\cdot\left(\frac{\frac{0.5\cdot[\text{ALA}]}{18.9}+\frac{0.5\cdot[\text{H2O\{CAC\}}]}{1}+\frac{0.5\cdot[\text{"NAD+"}]}{0.23}-\frac{0.4\cdot[\text{PYR}]}{1.7}+\frac{0.4\cdot[\text{NADH}]}{0.01}+\frac{0.4\cdot[\text{NH3}]}{5}}{1+\frac{[\text{ALA}]}{18.9}+\frac{[\text{H2O\{CAC\}}]}{1}+\frac{[\text{"NAD+"}]}{0.23}+\frac{[\text{PYR}]}{1.7}+\frac{[\text{NADH}]}{0.01}+\frac{[\text{NH3}]}{5}}\right)$$

$$\frac{d([\text{ATP\{CAC\}}]\cdot V_{CAC})}{dt} = +V_{CAC}\cdot\left(\frac{\frac{0.5\cdot[\text{ADP\{CAC\}}]}{3}+\frac{0.5\cdot[\text{ACP\{CAC\}}]}{0.024}-\frac{0.4\cdot[\text{ATP\{CAC\}}]}{0.16}+\frac{0.4\cdot[\text{ACE\{CAC\}}]}{40}}{1+\frac{[\text{ADP\{CAC\}}]}{3}+\frac{[\text{ACP\{CAC\}}]}{0.024}+\frac{[\text{ATP\{CAC\}}]}{0.16}+\frac{[\text{ACE\{CAC\}}]}{40}}\right)$$

$$\frac{d([\text{CO2\{CAC\}}]\cdot V_{CAC})}{dt} = +V_{CAC}\cdot\left(\frac{\frac{0.5\cdot[\text{PYR}]}{1.7}+\frac{0.5\cdot[\text{COA\{CAC\}}]}{0.037}+\frac{0.5\cdot[\text{"NAD+"}]}{1}-\frac{0.4\cdot[\text{ACA\{CAC\}}]}{2}+\frac{0.4\cdot[\text{CO2\{CAC\}}]}{10}+\frac{0.4\cdot[\text{NADH}]}{0.025}+\frac{0.4\cdot[\text{"H+\{CAC\}"}]}{10}}{1+\frac{[\text{PYR}]}{1.7}+\frac{[\text{COA\{CAC\}}]}{0.037}+\frac{[\text{"NAD+"}]}{1}+\frac{[\text{ACA\{CAC\}}]}{2}+\frac{[\text{CO2\{CAC\}}]}{10}+\frac{[\text{NADH}]}{0.025}+\frac{[\text{"H+\{CAC\}"}]}{10}}\right)$$

$$\frac{d([\text{COA\{CAC\}}]\cdot V_{CAC})}{dt} = -V_{CAC}\cdot\left(\frac{\frac{0.5\cdot[\text{PYR}]}{1.7}+\frac{0.5\cdot[\text{COA\{CAC\}}]}{0.037}+\frac{0.5\cdot[\text{"NAD+"}]}{1}-\frac{0.4\cdot[\text{ACA\{CAC\}}]}{2}+\frac{0.4\cdot[\text{CO2\{CAC\}}]}{10}+\frac{0.4\cdot[\text{NADH}]}{0.025}+\frac{0.4\cdot[\text{"H+\{CAC\}"}]}{10}}{1+\frac{[\text{PYR}]}{1.7}+\frac{[\text{COA\{CAC\}}]}{0.037}+\frac{[\text{"NAD+"}]}{1}+\frac{[\text{ACA\{CAC\}}]}{2}+\frac{[\text{CO2\{CAC\}}]}{10}+\frac{[\text{NADH}]}{0.025}+\frac{[\text{"H+\{CAC\}"}]}{10}}\right)$$
$$+V_{CAC}\cdot\left(\frac{\frac{0.5\cdot[\text{ACA\{CAC\}}]}{2}+\frac{0.5\cdot[\text{PO4\{CAC\}}]}{2.8}-\frac{0.4\cdot[\text{COA\{CAC\}}]}{0.03}+\frac{0.4\cdot[\text{ACP\{CAC\}}]}{89}}{1+\frac{[\text{ACA\{CAC\}}]}{2}+\frac{[\text{PO4\{CAC\}}]}{2.8}+\frac{[\text{COA\{CAC\}}]}{0.03}+\frac{[\text{ACP\{CAC\}}]}{89}}\right)$$

$$\frac{d([\text{GLY}]\cdot V_{CAC})}{dt} = -V_{CAC}\cdot\left(\frac{\frac{0.5\cdot[\text{GLY}]}{5.8}+\frac{0.5\cdot[\text{H2O\{CAC\}}]}{1}+\frac{0.5\cdot[\text{NADH}]}{0.1}-\frac{0.4\cdot[\text{PYR}]}{1.7}+\frac{0.4\cdot[\text{"NAD+"}]}{0.23}+\frac{0.4\cdot[\text{NH3}]}{5}}{1+\frac{[\text{GLY}]}{5.8}+\frac{[\text{H2O\{CAC\}}]}{1}+\frac{[\text{NADH}]}{0.1}+\frac{[\text{PYR}]}{1.7}+\frac{[\text{"NAD+"}]}{0.23}+\frac{[\text{NH3}]}{5}}\right)$$

$$-V_{CAC}\cdot\left(\frac{\frac{0.5\cdot[GLY]}{5.8}+\frac{0.5\cdot[PO4\{CAC\}]}{2.8}+\frac{0.5\cdot[TRD]}{1.69}-\frac{0.4\cdot[ACP\{CAC\}]}{0.032}+\frac{0.4\cdot[NH3]}{5}+\frac{0.4\cdot[RTRD]}{0.08}+\frac{0.4\cdot[H2O\{CAC\}]}{1}}{1+\frac{[GLY]}{5.8}+\frac{[H2O\{CAC\}]}{1}+\frac{[PO4\{CAC\}]}{2.8}+\frac{[TRD]}{1.69}+\frac{[ACP\{CAC\}]}{0.032}+\frac{[NH3]}{5}+\frac{[RTRD]}{0.08}}\right)$$

$$\frac{d(["H+\{CAC\}"]\cdot V_{CAC})}{dt}=+V_{CAC}\cdot\left(\frac{\frac{0.5\cdot[PYR]}{1.7}+\frac{0.5\cdot[COA\{CAC\}]}{0.037}+\frac{0.5\cdot["NAD+"]}{1}-\frac{0.4\cdot[ACA\{CAC\}]}{2}+\frac{0.4\cdot[CO2\{CAC\}]}{10}+\frac{0.4\cdot[NADH]}{0.025}+\frac{0.4\cdot["H+\{CAC\}"]}{10}}{1+\frac{[PYR]}{1.7}+\frac{[COA\{CAC\}]}{0.037}+\frac{["NAD+"]}{1}+\frac{[ACA\{CAC\}]}{2}+\frac{[CO2\{CAC\}]}{10}+\frac{[NADH]}{0.025}+\frac{["H+\{CAC\}"]}{10}}\right)$$

$$\frac{d([H2O\{CAC\}]\cdot V_{CAC})}{dt}=-V_{CAC}\cdot\left(\frac{\frac{0.5\cdot[ALA]}{18.9}+\frac{0.5\cdot[H2O\{CAC\}]}{1}+\frac{0.5\cdot["NAD+"]}{0.23}-\frac{0.4\cdot[PYR]}{1.7}+\frac{0.4\cdot[NADH]}{0.01}+\frac{0.4\cdot[NH3]}{5}}{1+\frac{[ALA]}{18.9}+\frac{[H2O\{CAC\}]}{1}+\frac{["NAD+"]}{0.23}+\frac{[PYR]}{1.7}+\frac{[NADH]}{0.01}+\frac{[NH3]}{5}}\right)$$

$$-V_{CAC}\cdot\left(\frac{\frac{0.5\cdot[GLY]}{5.8}+\frac{0.5\cdot[H2O\{CAC\}]}{1}+\frac{0.5\cdot[NADH]}{0.1}-\frac{0.4\cdot[PYR]}{1.7}+\frac{0.4\cdot["NAD+"]}{0.23}+\frac{0.4\cdot[NH3]}{5}}{1+\frac{[GLY]}{5.8}+\frac{[H2O\{CAC\}]}{1}+\frac{[NADH]}{0.1}+\frac{[PYR]}{1.7}+\frac{["NAD+"]}{0.23}+\frac{[NH3]}{5}}\right)$$

$$+V_{CAC}\cdot\left(\frac{\frac{0.5\cdot[GLY]}{5.8}+\frac{0.5\cdot[PO4\{CAC\}]}{2.8}+\frac{0.5\cdot[TRD]}{1.69}-\frac{0.4\cdot[ACP\{CAC\}]}{0.032}+\frac{0.4\cdot[NH3]}{5}+\frac{0.4\cdot[RTRD]}{0.08}+\frac{0.4\cdot[H2O\{CAC\}]}{1}}{1+\frac{[GLY]}{5.8}+\frac{[H2O\{CAC\}]}{1}+\frac{[PO4\{CAC\}]}{2.8}+\frac{[TRD]}{1.69}+\frac{[ACP\{CAC\}]}{0.032}+\frac{[NH3]}{5}+\frac{[RTRD]}{0.08}}\right)$$

$$\frac{d(["NAD+"]\cdot V_{CAC})}{dt}=-V_{CAC}\cdot\left(\frac{\frac{0.5\cdot[ALA]}{18.9}+\frac{0.5\cdot[H2O\{CAC\}]}{1}+\frac{0.5\cdot["NAD+"]}{0.23}-\frac{0.4\cdot[PYR]}{1.7}+\frac{0.4\cdot[NADH]}{0.01}+\frac{0.4\cdot[NH3]}{5}}{1+\frac{[ALA]}{18.9}+\frac{[H2O\{CAC\}]}{1}+\frac{["NAD+"]}{0.23}+\frac{[PYR]}{1.7}+\frac{[NADH]}{0.01}+\frac{[NH3]}{5}}\right)$$

$$+V_{CAC}\cdot\left(\frac{\frac{0.5\cdot[GLY]}{5.8}+\frac{0.5\cdot[H2O\{CAC\}]}{1}+\frac{0.5\cdot[NADH]}{0.1}-\frac{0.4\cdot[PYR]}{1.7}+\frac{0.4\cdot["NAD+"]}{0.23}+\frac{0.4\cdot[NH3]}{5}}{1+\frac{[GLY]}{5.8}+\frac{[H2O\{CAC\}]}{1}+\frac{[NADH]}{0.1}+\frac{[PYR]}{1.7}+\frac{["NAD+"]}{0.23}+\frac{[NH3]}{5}}\right)$$

$$-V_{CAC}\cdot\left(\frac{\frac{0.5\cdot[PYR]}{1.7}+\frac{0.5\cdot[COA\{CAC\}]}{0.037}+\frac{0.5\cdot["NAD+"]}{1}-\frac{0.4\cdot[ACA\{CAC\}]}{2}+\frac{0.4\cdot[CO2\{CAC\}]}{10}+\frac{0.4\cdot[NADH]}{0.025}+\frac{0.4\cdot["H+\{CAC\}"]}{10}}{1+\frac{[PYR]}{1.7}+\frac{[COA\{CAC\}]}{0.037}+\frac{["NAD+"]}{1}+\frac{[ACA\{CAC\}]}{2}+\frac{[CO2\{CAC\}]}{10}+\frac{[NADH]}{0.025}+\frac{["H+\{CAC\}"]}{10}}\right)$$

$$\frac{d([NADH]\cdot V_{CAC})}{dt}=+V_{CAC}\cdot\left(\frac{\frac{0.5\cdot[ALA]}{18.9}+\frac{0.5\cdot[H2O\{CAC\}]}{1}+\frac{0.5\cdot["NAD+"]}{0.23}-\frac{0.4\cdot[PYR]}{1.7}+\frac{0.4\cdot[NADH]}{0.01}+\frac{0.4\cdot[NH3]}{5}}{1+\frac{[ALA]}{18.9}+\frac{[H2O\{CAC\}]}{1}+\frac{["NAD+"]}{0.23}+\frac{[PYR]}{1.7}+\frac{[NADH]}{0.01}+\frac{[NH3]}{5}}\right)$$

$$-V_{CAC}\cdot\left(\frac{\frac{0.5\cdot[GLY]}{5.8}+\frac{0.5\cdot[H2O\{CAC\}]}{1}+\frac{0.5\cdot[NADH]}{0.1}-\frac{0.4\cdot[PYR]}{1.7}+\frac{0.4\cdot["NAD+"]}{0.23}+\frac{0.4\cdot[NH3]}{5}}{1+\frac{[GLY]}{5.8}+\frac{[H2O\{CAC\}]}{1}+\frac{[NADH]}{0.1}+\frac{[PYR]}{1.7}+\frac{["NAD+"]}{0.23}+\frac{[NH3]}{5}}\right)$$

$$+V_{CAC}\cdot\left(\frac{\frac{0.5\cdot[PYR]}{1.7}+\frac{0.5\cdot[COA\{CAC\}]}{0.037}+\frac{0.5\cdot["NAD+"]}{1}-\frac{0.4\cdot[ACA\{CAC\}]}{2}+\frac{0.4\cdot[CO2\{CAC\}]}{10}+\frac{0.4\cdot[NADH]}{0.025}+\frac{0.4\cdot["H+\{CAC\}"]}{10}}{1+\frac{[PYR]}{1.7}+\frac{[COA\{CAC\}]}{0.037}+\frac{["NAD+"]}{1}+\frac{[ACA\{CAC\}]}{2}+\frac{[CO2\{CAC\}]}{10}+\frac{[NADH]}{0.025}+\frac{["H+\{CAC\}"]}{10}}\right)$$

$$\frac{d([NH3]\cdot V_{CAC})}{dt}=+V_{CAC}\cdot\left(\frac{\frac{0.5\cdot[ALA]}{18.9}+\frac{0.5\cdot[H2O\{CAC\}]}{1}+\frac{0.5\cdot["NAD+"]}{0.23}-\frac{0.4\cdot[PYR]}{1.7}+\frac{0.4\cdot[NADH]}{0.01}+\frac{0.4\cdot[NH3]}{5}}{1+\frac{[ALA]}{18.9}+\frac{[H2O\{CAC\}]}{1}+\frac{["NAD+"]}{0.23}+\frac{[PYR]}{1.7}+\frac{[NADH]}{0.01}+\frac{[NH3]}{5}}\right)$$

$$+V_{CAC}\cdot\left(\frac{\frac{0.5\cdot[GLY]}{5.8}+\frac{0.5\cdot[H2O\{CAC\}]}{1}+\frac{0.5\cdot[NADH]}{0.1}-\frac{0.4\cdot[PYR]}{1.7}+\frac{0.4\cdot["NAD+"]}{0.23}+\frac{0.4\cdot[NH3]}{5}}{1+\frac{[GLY]}{5.8}+\frac{[H2O\{CAC\}]}{1}+\frac{[NADH]}{0.1}+\frac{[PYR]}{1.7}+\frac{["NAD+"]}{0.23}+\frac{[NH3]}{5}}\right)$$

$$+V_{CAC}\cdot\left(\frac{\frac{0.5\cdot[GLY]}{5.8}+\frac{0.5\cdot[PO4\{CAC\}]}{2.8}+\frac{0.5\cdot[TRD]}{1.69}-\frac{0.4\cdot[ACP\{CAC\}]}{0.032}+\frac{0.4\cdot[NH3]}{5}+\frac{0.4\cdot[RTRD]}{0.08}+\frac{0.4\cdot[H2O\{CAC\}]}{1}}{1+\frac{[GLY]}{5.8}+\frac{[H2O\{CAC\}]}{1}+\frac{[PO4\{CAC\}]}{2.8}+\frac{[TRD]}{1.69}+\frac{[ACP\{CAC\}]}{0.032}+\frac{[NH3]}{5}+\frac{[RTRD]}{0.08}}\right)$$

$$\frac{d([PO4\{CAC\}]\cdot V_{CAC})}{dt}=-V_{CAC}\cdot\left(\frac{\frac{0.5\cdot[GLY]}{5.8}+\frac{0.5\cdot[PO4\{CAC\}]}{2.8}+\frac{0.5\cdot[TRD]}{1.69}-\frac{0.4\cdot[ACP\{CAC\}]}{0.032}+\frac{0.4\cdot[NH3]}{5}+\frac{0.4\cdot[RTRD]}{0.08}+\frac{0.4\cdot[H2O\{CAC\}]}{1}}{1+\frac{[GLY]}{5.8}+\frac{[H2O\{CAC\}]}{1}+\frac{[PO4\{CAC\}]}{2.8}+\frac{[TRD]}{1.69}+\frac{[ACP\{CAC\}]}{0.032}+\frac{[NH3]}{5}+\frac{[RTRD]}{0.08}}\right)$$

$$-V_{CAC}\cdot\left(\frac{\frac{0.5\cdot[ACA\{CAC\}]}{2}+\frac{0.5\cdot[PO4\{CAC\}]}{2.8}-\frac{0.4\cdot[COA\{CAC\}]}{0.03}+\frac{0.4\cdot[ACP\{CAC\}]}{89}}{1+\frac{[ACA\{CAC\}]}{2}+\frac{[PO4\{CAC\}]}{2.8}+\frac{[COA\{CAC\}]}{0.03}+\frac{[ACP\{CAC\}]}{89}}\right)$$

$$\frac{d([PYR]\cdot V_{CAC})}{dt}=+V_{CAC}\cdot\left(\frac{\frac{0.5\cdot[ALA]}{18.9}+\frac{0.5\cdot[H2O\{CAC\}]}{1}+\frac{0.5\cdot["NAD+"]}{0.23}-\frac{0.4\cdot[PYR]}{1.7}+\frac{0.4\cdot[NADH]}{0.01}+\frac{0.4\cdot[NH3]}{5}}{1+\frac{[ALA]}{18.9}+\frac{[H2O\{CAC\}]}{1}+\frac{["NAD+"]}{0.23}+\frac{[PYR]}{1.7}+\frac{[NADH]}{0.01}+\frac{[NH3]}{5}}\right)$$

$$+V_{CAC}\cdot\left(\frac{\frac{0.5\cdot[GLY]}{5.8}+\frac{0.5\cdot[H2O\{CAC\}]}{1}+\frac{0.5\cdot[NADH]}{0.1}-\frac{0.4\cdot[PYR]}{1.7}+\frac{0.4\cdot["NAD+"]}{0.23}+\frac{0.4\cdot[NH3]}{5}}{1+\frac{[GLY]}{5.8}+\frac{[H2O\{CAC\}]}{1}+\frac{[NADH]}{0.1}+\frac{[PYR]}{1.7}+\frac{["NAD+"]}{0.23}+\frac{[NH3]}{5}}\right)$$

$$
-V_{CAC}\cdot\left(\frac{\frac{0.5\cdot[\text{PYR}]}{1.7}+\frac{0.5\cdot[\text{COA\{CAC\}}]}{0.037}+\frac{0.5\cdot[\text{"NAD+"}]}{1}-\frac{0.4\cdot[\text{ACA\{CAC\}}]}{2}+\frac{0.4\cdot[\text{CO2\{CAC\}}]}{10}+\frac{0.4\cdot[\text{NADH}]}{0.025}+\frac{0.4\cdot[\text{"H+\{CAC\}"}]}{10}}{1+\frac{[\text{PYR}]}{1.7}+\frac{[\text{COA\{CAC\}}]}{0.037}+\frac{[\text{"NAD+"}]}{1}+\frac{[\text{ACA\{CAC\}}]}{2}+\frac{[\text{CO2\{CAC\}}]}{10}+\frac{[\text{NADH}]}{0.025}+\frac{[\text{"H+\{CAC\}"}]}{10}}\right)
$$

$$
\frac{d([\text{RTRD}]\cdot V_{CAC})}{dt}=+V_{CAC}\cdot\left(\frac{\frac{0.5\cdot[\text{GLY}]}{5.8}+\frac{0.5\cdot[\text{PO4\{CAC\}}]}{2.8}+\frac{0.5\cdot[\text{TRD}]}{1.69}-\frac{0.4\cdot[\text{ACP\{CAC\}}]}{0.032}+\frac{0.4\cdot[\text{NH3}]}{5}+\frac{0.4\cdot[\text{RTRD}]}{0.08}+\frac{0.4\cdot[\text{H2O\{CAC\}}]}{1}}{1+\frac{[\text{GLY}]}{5.8}+\frac{[\text{H2O\{CAC\}}]}{1}+\frac{[\text{PO4\{CAC\}}]}{2.8}+\frac{[\text{TRD}]}{1.69}+\frac{[\text{ACP\{CAC\}}]}{0.032}+\frac{[\text{NH3}]}{5}+\frac{[\text{RTRD}]}{0.08}}\right)
$$

$$
\frac{d([\text{TRD}]\cdot V_{CAC})}{dt}=-V_{CAC}\cdot\left(\frac{\frac{0.5\cdot[\text{GLY}]}{5.8}+\frac{0.5\cdot[\text{PO4\{CAC\}}]}{2.8}+\frac{0.5\cdot[\text{TRD}]}{1.69}-\frac{0.4\cdot[\text{ACP\{CAC\}}]}{0.032}+\frac{0.4\cdot[\text{NH3}]}{5}+\frac{0.4\cdot[\text{RTRD}]}{0.08}+\frac{0.4\cdot[\text{H2O\{CAC\}}]}{1}}{1+\frac{[\text{GLY}]}{5.8}+\frac{[\text{H2O\{CAC\}}]}{1}+\frac{[\text{PO4\{CAC\}}]}{2.8}+\frac{[\text{TRD}]}{1.69}+\frac{[\text{ACP\{CAC\}}]}{0.032}+\frac{[\text{NH3}]}{5}+\frac{[\text{RTRD}]}{0.08}}\right)
$$

$$
\frac{d([\text{ACA\{MAC\}}]\cdot V_{MAC})}{dt}=+V_{MAC}\cdot\left(\frac{\frac{0.5\cdot[\text{ATP\{MAC\}}]}{0.133}+\frac{0.5\cdot[\text{ACE\{MAC\}}]}{0.258}+\frac{0.5\cdot[\text{COA\{MAC\}}]}{0.027}-\frac{0.4\cdot[\text{ADP\{MAC\}}]}{1}+\frac{0.4\cdot[\text{PO4\{MAC\}}]}{0.742}+\frac{0.4\cdot[\text{ACA\{MAC\}}]}{0.28}}{1+\frac{[\text{ATP\{MAC\}}]}{0.133}+\frac{[\text{ACE\{MAC\}}]}{0.258}+\frac{[\text{COA\{MAC\}}]}{0.027}+\frac{[\text{ADP\{MAC\}}]}{1}+\frac{[\text{PO4\{MAC\}}]}{0.742}+\frac{[\text{ACA\{MAC\}}]}{0.28}}\right)
$$

$$
+V_{MAC}\cdot\left(\frac{\frac{0.5\cdot[\text{ACP\{MAC\}}]}{15}+\frac{0.5\cdot[\text{COA\{MAC\}}]}{0.034}-\frac{0.4\cdot[\text{ACA\{MAC\}}]}{0.023}+\frac{0.4\cdot[\text{PO4\{MAC\}}]}{2.8}}{1+\frac{[\text{ACA\{MAC\}}]}{0.023}+\frac{[\text{ACP\{MAC\}}]}{15}+\frac{[\text{COA\{MAC\}}]}{0.034}+\frac{[\text{PO4\{MAC\}}]}{2.8}}\right)
$$

$$
-V_{MAC}\cdot\left(\frac{\frac{0.5\cdot[\text{ACA\{MAC\}}]}{0.28}+\frac{0.5\cdot[\text{CFP}]}{0.1}-\frac{0.1\cdot[\text{COA\{MAC\}}]}{4.3}+\frac{0.1\cdot[\text{CO}]}{0.053}+\frac{0.1\cdot[\text{MCP}]}{0.012}}{1+\frac{[\text{ACA\{MAC\}}]}{0.28}+\frac{[\text{CFP}]}{0.1}+\frac{[\text{COA\{MAC\}}]}{4.3}+\frac{[\text{CO}]}{0.053}+\frac{[\text{MCP}]}{0.012}}\right)
$$

$$
\frac{d([\text{ACE\{MAC\}}]\cdot V_{MAC})}{dt}=+([\text{ACE\{CAC\}}]\cdot 0.1)
$$

$$
-V_{MAC}\cdot\left(\frac{\frac{0.5\cdot[\text{ATP\{MAC\}}]}{2.8}+\frac{0.5\cdot[\text{ACE\{MAC\}}]}{0.258}-\frac{0.4\cdot[\text{ADP\{MAC\}}]}{1}+\frac{0.4\cdot[\text{ACP\{MAC\}}]}{89}}{1+\frac{[\text{ADP\{MAC\}}]}{1}+\frac{[\text{ACP\{MAC\}}]}{89}+\frac{[\text{ATP\{MAC\}}]}{2.8}+\frac{[\text{ACE\{MAC\}}]}{0.258}}\right)
$$

$$
-V_{MAC}\cdot\left(\frac{\frac{0.5\cdot[\text{ATP\{MAC\}}]}{0.133}+\frac{0.5\cdot[\text{ACE\{MAC\}}]}{0.258}+\frac{0.5\cdot[\text{COA\{MAC\}}]}{0.027}-\frac{0.4\cdot[\text{ADP\{MAC\}}]}{1}+\frac{0.4\cdot[\text{PO4\{MAC\}}]}{0.742}+\frac{0.4\cdot[\text{ACA\{MAC\}}]}{0.28}}{1+\frac{[\text{ATP\{MAC\}}]}{0.133}+\frac{[\text{ACE\{MAC\}}]}{0.258}+\frac{[\text{COA\{MAC\}}]}{0.027}+\frac{[\text{ADP\{MAC\}}]}{1}+\frac{[\text{PO4\{MAC\}}]}{0.742}+\frac{[\text{ACA\{MAC\}}]}{0.28}}\right)
$$

$$
\frac{d([\text{ACP\{MAC\}}]\cdot V_{MAC})}{dt}=+V_{MAC}\cdot\left(\frac{\frac{0.5\cdot[\text{ATP\{MAC\}}]}{2.8}+\frac{0.5\cdot[\text{ACE\{MAC\}}]}{0.258}-\frac{0.4\cdot[\text{ADP\{MAC\}}]}{1}+\frac{0.4\cdot[\text{ACP\{MAC\}}]}{89}}{1+\frac{[\text{ADP\{MAC\}}]}{1}+\frac{[\text{ACP\{MAC\}}]}{89}+\frac{[\text{ATP\{MAC\}}]}{2.8}+\frac{[\text{ACE\{MAC\}}]}{0.258}}\right)
$$

$$
-V_{MAC}\cdot\left(\frac{\frac{0.5\cdot[\text{ACP\{MAC\}}]}{15}+\frac{0.5\cdot[\text{COA\{MAC\}}]}{0.034}-\frac{0.4\cdot[\text{ACA\{MAC\}}]}{0.023}+\frac{0.4\cdot[\text{PO4\{MAC\}}]}{2.8}}{1+\frac{[\text{ACA\{MAC\}}]}{0.023}+\frac{[\text{ACP\{MAC\}}]}{15}+\frac{[\text{COA\{MAC\}}]}{0.034}+\frac{[\text{PO4\{MAC\}}]}{2.8}}\right)
$$

$$
\frac{d([\text{ADP\{MAC\}}]\cdot V_{MAC})}{dt}=-V_{MAC}\cdot\left(\frac{0.5\cdot[\text{ADP\{MAC\}}]}{2.3225+[\text{ADP\{MAC\}}]}+\frac{0.5\cdot[\text{PO4\{MAC\}}]}{2.3225+[\text{PO4\{MAC\}}]}+\frac{0.5\cdot[\text{"H+\{MAC\}"}]}{2.3225+[\text{"H+\{MAC\}"}]}\right)
$$

$$
+V_{MAC}\cdot\left(\frac{\frac{0.5\cdot[\text{ATP\{MAC\}}]}{2.8}+\frac{0.5\cdot[\text{ACE\{MAC\}}]}{0.258}-\frac{0.4\cdot[\text{ADP\{MAC\}}]}{1}+\frac{0.4\cdot[\text{ACP\{MAC\}}]}{89}}{1+\frac{[\text{ADP\{MAC\}}]}{1}+\frac{[\text{ACP\{MAC\}}]}{89}+\frac{[\text{ATP\{MAC\}}]}{2.8}+\frac{[\text{ACE\{MAC\}}]}{0.258}}\right)
$$

$$
+V_{MAC}\cdot\left(\frac{\frac{0.5\cdot[\text{ATP\{MAC\}}]}{0.133}+\frac{0.5\cdot[\text{ACE\{MAC\}}]}{0.258}+\frac{0.5\cdot[\text{COA\{MAC\}}]}{0.027}-\frac{0.4\cdot[\text{ADP\{MAC\}}]}{1}+\frac{0.4\cdot[\text{PO4\{MAC\}}]}{0.742}+\frac{0.4\cdot[\text{ACA\{MAC\}}]}{0.28}}{1+\frac{[\text{ATP\{MAC\}}]}{0.133}+\frac{[\text{ACE\{MAC\}}]}{0.258}+\frac{[\text{COA\{MAC\}}]}{0.027}+\frac{[\text{ADP\{MAC\}}]}{1}+\frac{[\text{PO4\{MAC\}}]}{0.742}+\frac{[\text{ACA\{MAC\}}]}{0.28}}\right)
$$

$$
\begin{aligned}
\frac{d([\mathrm{ATP\{MAC\}}]\cdot V_{MAC})}{dt} = {} & +V_{MAC}\cdot\left(\frac{0.5\cdot[\mathrm{ADP\{MAC\}}]}{2.3225+[\mathrm{ADP\{MAC\}}]}+\frac{0.5\cdot[\mathrm{PO4\{MAC\}}]}{2.3225+[\mathrm{PO4\{MAC\}}]}+\frac{0.5\cdot[\mathrm{"H+\{MAC\}"}]}{2.3225+[\mathrm{"H+\{MAC\}"}]}\right)\\
& -V_{MAC}\cdot\left(\frac{\frac{0.5\cdot[\mathrm{ATP\{MAC\}}]}{2.8}+\frac{0.5\cdot[\mathrm{ACE\{MAC\}}]}{0.258}-\frac{0.4\cdot[\mathrm{ADP\{MAC\}}]}{1}+\frac{0.4\cdot[\mathrm{ACP\{MAC\}}]}{89}}{1+\frac{[\mathrm{ADP\{MAC\}}]}{1}+\frac{[\mathrm{ACP\{MAC\}}]}{89}+\frac{[\mathrm{ATP\{MAC\}}]}{2.8}+\frac{[\mathrm{ACE\{MAC\}}]}{0.258}}\right)\\
& -V_{MAC}\cdot\left(\frac{\frac{0.5\cdot[\mathrm{ATP\{MAC\}}]}{0.133}+\frac{0.5\cdot[\mathrm{ACE\{MAC\}}]}{0.258}+\frac{0.5\cdot[\mathrm{COA\{MAC\}}]}{0.027}-\frac{0.4\cdot[\mathrm{ADP\{MAC\}}]}{1}+\frac{0.4\cdot[\mathrm{PO4\{MAC\}}]}{0.742}+\frac{0.4\cdot[\mathrm{ACA\{MAC\}}]}{0.28}}{1+\frac{[\mathrm{ATP\{MAC\}}]}{0.133}+\frac{[\mathrm{ACE\{MAC\}}]}{0.258}+\frac{[\mathrm{COA\{MAC\}}]}{0.027}+\frac{[\mathrm{ADP\{MAC\}}]}{1}+\frac{[\mathrm{PO4\{MAC\}}]}{0.742}+\frac{[\mathrm{ACA\{MAC\}}]}{0.28}}\right)
\end{aligned}
$$

$$
\begin{aligned}
\frac{d([\mathrm{CFP}]\cdot V_{MAC})}{dt} = {} & +V_{MAC}\cdot\left(\frac{\frac{0.5\cdot[\mathrm{MCP}]}{0.012}+\frac{0.5\cdot[\mathrm{THM}]}{0.002}-\frac{0.4\cdot[\mathrm{CFP}]}{0.002}+\frac{0.4\cdot[\mathrm{MTHM}]}{0.0756}}{1+\frac{[\mathrm{MCP}]}{0.012}+\frac{[\mathrm{THM}]}{0.002}+\frac{[\mathrm{CFP}]}{0.002}+\frac{[\mathrm{MTHM}]}{0.0756}}\right)\\
& -V_{MAC}\cdot\left(\frac{\frac{0.5\cdot[\mathrm{ACA\{MAC\}}]}{0.28}+\frac{0.5\cdot[\mathrm{CFP}]}{0.1}-\frac{0.1\cdot[\mathrm{COA\{MAC\}}]}{4.3}+\frac{0.1\cdot[\mathrm{CO}]}{0.053}+\frac{0.1\cdot[\mathrm{MCP}]}{0.012}}{1+\frac{[\mathrm{ACA\{MAC\}}]}{0.28}+\frac{[\mathrm{CFP}]}{0.1}+\frac{[\mathrm{COA\{MAC\}}]}{4.3}+\frac{[\mathrm{CO}]}{0.053}+\frac{[\mathrm{MCP}]}{0.012}}\right)
\end{aligned}
$$

$$
\frac{d([\mathrm{CH4}]\cdot V_{MAC})}{dt} = +V_{MAC}\cdot\left(\frac{\frac{0.5\cdot[\mathrm{MCOM}]}{0.6}+\frac{0.5\cdot[\mathrm{COB}]}{0.1}-\frac{0.1\cdot[\mathrm{COMB}]}{1.8}+\frac{0.1\cdot[\mathrm{CH4}]}{0.066}}{1+\frac{[\mathrm{MCOM}]}{0.6}+\frac{[\mathrm{COB}]}{0.1}+\frac{[\mathrm{COMB}]}{1.8}+\frac{[\mathrm{CH4}]}{0.066}}\right)
$$

$$
\begin{aligned}
\frac{d([\mathrm{CO}]\cdot V_{MAC})}{dt} = {} & -V_{MAC}\cdot\left(\frac{0.5\cdot[\mathrm{CO}]}{0.1553+[\mathrm{CO}]}+\frac{0.5\cdot[\mathrm{H2O\{MAC\}}]}{0.1553+[\mathrm{H2O\{MAC\}}]}+\frac{0.5\cdot[\mathrm{OFD}]}{0.1553+[\mathrm{OFD}]}\right)\\
& +V_{MAC}\cdot\left(\frac{\frac{0.5\cdot[\mathrm{ACA\{MAC\}}]}{0.28}+\frac{0.5\cdot[\mathrm{CFP}]}{0.1}-\frac{0.1\cdot[\mathrm{COA\{MAC\}}]}{4.3}+\frac{0.1\cdot[\mathrm{CO}]}{0.053}+\frac{0.1\cdot[\mathrm{MCP}]}{0.012}}{1+\frac{[\mathrm{ACA\{MAC\}}]}{0.28}+\frac{[\mathrm{CFP}]}{0.1}+\frac{[\mathrm{COA\{MAC\}}]}{4.3}+\frac{[\mathrm{CO}]}{0.053}+\frac{[\mathrm{MCP}]}{0.012}}\right)
\end{aligned}
$$

$$
\frac{d([\mathrm{CO2\{MAC\}}]\cdot V_{MAC})}{dt} = +V_{MAC}\cdot\left(\frac{0.5\cdot[\mathrm{CO}]}{0.1553+[\mathrm{CO}]}+\frac{0.5\cdot[\mathrm{H2O\{MAC\}}]}{0.1553+[\mathrm{H2O\{MAC\}}]}+\frac{0.5\cdot[\mathrm{OFD}]}{0.1553+[\mathrm{OFD}]}\right)
$$

$$
\begin{aligned}
\frac{d([\mathrm{COA\{MAC\}}]\cdot V_{MAC})}{dt} = {} & -V_{MAC}\cdot\left(\frac{\frac{0.5\cdot[\mathrm{ATP\{MAC\}}]}{0.133}+\frac{0.5\cdot[\mathrm{ACE\{MAC\}}]}{0.258}+\frac{0.5\cdot[\mathrm{COA\{MAC\}}]}{0.027}-\frac{0.4\cdot[\mathrm{ADP\{MAC\}}]}{1}+\frac{0.4\cdot[\mathrm{PO4\{MAC\}}]}{0.742}+\frac{0.4\cdot[\mathrm{ACA\{MAC\}}]}{0.28}}{1+\frac{[\mathrm{ATP\{MAC\}}]}{0.133}+\frac{[\mathrm{ACE\{MAC\}}]}{0.258}+\frac{[\mathrm{COA\{MAC\}}]}{0.027}+\frac{[\mathrm{ADP\{MAC\}}]}{1}+\frac{[\mathrm{PO4\{MAC\}}]}{0.742}+\frac{[\mathrm{ACA\{MAC\}}]}{0.28}}\right)\\
& -V_{MAC}\cdot\left(\frac{\frac{0.5\cdot[\mathrm{ACP\{MAC\}}]}{15}+\frac{0.5\cdot[\mathrm{COA\{MAC\}}]}{0.034}-\frac{0.4\cdot[\mathrm{ACA\{MAC\}}]}{0.023}+\frac{0.4\cdot[\mathrm{PO4\{MAC\}}]}{2.8}}{1+\frac{[\mathrm{ACA\{MAC\}}]}{0.023}+\frac{[\mathrm{ACP\{MAC\}}]}{15}+\frac{[\mathrm{COA\{MAC\}}]}{0.034}+\frac{[\mathrm{PO4\{MAC\}}]}{2.8}}\right)\\
& +V_{MAC}\cdot\left(\frac{\frac{0.5\cdot[\mathrm{ACA\{MAC\}}]}{0.28}+\frac{0.5\cdot[\mathrm{CFP}]}{0.1}-\frac{0.1\cdot[\mathrm{COA\{MAC\}}]}{4.3}+\frac{0.1\cdot[\mathrm{CO}]}{0.053}+\frac{0.1\cdot[\mathrm{MCP}]}{0.012}}{1+\frac{[\mathrm{ACA\{MAC\}}]}{0.28}+\frac{[\mathrm{CFP}]}{0.1}+\frac{[\mathrm{COA\{MAC\}}]}{4.3}+\frac{[\mathrm{CO}]}{0.053}+\frac{[\mathrm{MCP}]}{0.012}}\right)
\end{aligned}
$$

$$
\begin{aligned}
\frac{d([\mathrm{COB}]\cdot V_{MAC})}{dt} = {} & -V_{MAC}\cdot\left(\frac{\frac{0.5\cdot[\mathrm{MCOM}]}{0.6}+\frac{0.5\cdot[\mathrm{COB}]}{0.1}-\frac{0.1\cdot[\mathrm{COMB}]}{1.8}+\frac{0.1\cdot[\mathrm{CH4}]}{0.066}}{1+\frac{[\mathrm{MCOM}]}{0.6}+\frac{[\mathrm{COB}]}{0.1}+\frac{[\mathrm{COMB}]}{1.8}+\frac{[\mathrm{CH4}]}{0.066}}\right)\\
& +V_{MAC}\cdot\left(\frac{\frac{0.5\cdot[\mathrm{COMB}]}{0.1}+\frac{0.5\cdot[\mathrm{MPH2}]}{0.092}-\frac{0.4\cdot[\mathrm{COM}]}{0.8}+\frac{0.4\cdot[\mathrm{COB}]}{0.25}+\frac{0.4\cdot[\mathrm{"H+\{MAC\}"}]}{0.03}+\frac{0.4\cdot[\mathrm{MP}]}{0.25}}{1+\frac{[\mathrm{COMB}]}{0.1}+\frac{[\mathrm{COB}]}{0.25}+\frac{[\mathrm{MPH2}]}{0.092}+\frac{[\mathrm{COM}]}{0.8}+\frac{[\mathrm{"H+\{MAC\}"}]}{0.03}+\frac{[\mathrm{MP}]}{0.25}}\right)
\end{aligned}
$$

$$
\frac{d([\mathrm{COM}]\cdot V_{MAC})}{dt} = -V_{MAC}\cdot\left(\frac{\frac{0.5\cdot[\mathrm{MTHM}]}{0.5}+\frac{0.5\cdot[\mathrm{COM}]}{1}-\frac{0.4\cdot[\mathrm{THM}]}{3.7}+\frac{0.4\cdot[\mathrm{MCOM}]}{0.6}}{1+\frac{[\mathrm{MTHM}]}{0.5}+\frac{[\mathrm{COM}]}{1}+\frac{[\mathrm{MCOM}]}{0.6}+\frac{[\mathrm{THM}]}{3.7}}\right)
$$

$$+V_{MAC}\cdot\left(\frac{\frac{0.5\cdot[\text{COMB}]}{0.1}+\frac{0.5\cdot[\text{MPH2}]}{0.092}-\frac{0.4\cdot[\text{COM}]}{0.8}+\frac{0.4\cdot[\text{COB}]}{0.25}+\frac{0.4\cdot[\text{"H+\{MAC\}"}]}{0.03}+\frac{0.4\cdot[\text{MP}]}{0.25}}{1+\frac{[\text{COMB}]}{0.1}+\frac{[\text{COB}]}{0.25}+\frac{[\text{MPH2}]}{0.092}+\frac{[\text{COM}]}{0.8}+\frac{[\text{"H+\{MAC\}"}]}{0.03}+\frac{[\text{MP}]}{0.25}}\right)$$

$$\frac{d([\text{COMB}]\cdot V_{MAC})}{dt}=+V_{MAC}\cdot\left(\frac{\frac{0.5\cdot[\text{MCOM}]}{0.6}+\frac{0.5\cdot[\text{COB}]}{0.1}-\frac{0.1\cdot[\text{COMB}]}{1.8}+\frac{0.1\cdot[\text{CH4}]}{0.066}}{1+\frac{[\text{MCOM}]}{0.6}+\frac{[\text{COB}]}{0.1}+\frac{[\text{COMB}]}{1.8}+\frac{[\text{CH4}]}{0.066}}\right)$$

$$-V_{MAC}\cdot\left(\frac{\frac{0.5\cdot[\text{COMB}]}{0.1}+\frac{0.5\cdot[\text{MPH2}]}{0.092}-\frac{0.4\cdot[\text{COM}]}{0.8}+\frac{0.4\cdot[\text{COB}]}{0.25}+\frac{0.4\cdot[\text{"H+\{MAC\}"}]}{0.03}+\frac{0.4\cdot[\text{MP}]}{0.25}}{1+\frac{[\text{COMB}]}{0.1}+\frac{[\text{COB}]}{0.25}+\frac{[\text{MPH2}]}{0.092}+\frac{[\text{COM}]}{0.8}+\frac{[\text{"H+\{MAC\}"}]}{0.03}+\frac{[\text{MP}]}{0.25}}\right)$$

$$\frac{d([\text{F420}]\cdot V_{MAC})}{dt}=+V_{MAC}\cdot\left(\frac{\frac{0.5\cdot[\text{F420H2}]}{0.002}+\frac{0.5\cdot[\text{MP}]}{0.25}-\frac{0.1\cdot[\text{F420}]}{0.043}+\frac{0.1\cdot[\text{MPH2}]}{0.092}+\frac{0.1\cdot[\text{"H+\{MAC\}"}]}{10}}{1+\frac{[\text{F420H2}]}{0.002}+\frac{[\text{MPH2}]}{0.092}+\frac{[\text{MP}]}{0.25}+\frac{[\text{F420}]}{0.043}+\frac{[\text{"H+\{MAC\}"}]}{10}}\right)$$

$$\frac{d([\text{F420H2}]\cdot V_{MAC})}{dt}=-V_{MAC}\cdot\left(\frac{\frac{0.5\cdot[\text{F420H2}]}{0.002}+\frac{0.5\cdot[\text{MP}]}{0.25}-\frac{0.1\cdot[\text{F420}]}{0.043}+\frac{0.1\cdot[\text{MPH2}]}{0.092}+\frac{0.1\cdot[\text{"H+\{MAC\}"}]}{10}}{1+\frac{[\text{F420H2}]}{0.002}+\frac{[\text{MPH2}]}{0.092}+\frac{[\text{MP}]}{0.25}+\frac{[\text{F420}]}{0.043}+\frac{[\text{"H+\{MAC\}"}]}{10}}\right)$$

$$\frac{d([\text{"H+\{MAC\}"}]\cdot V_{MAC})}{dt}=+V_{MAC}\cdot\left(\frac{0.5\cdot[\text{CO}]}{0.1553+[\text{CO}]}+\frac{0.5\cdot[\text{H2O\{MAC\}}]}{0.1553+[\text{H2O\{MAC\}}]}+\frac{0.5\cdot[\text{OFD}]}{0.1553+[\text{OFD}]}\right)$$

$$+V_{MAC}\cdot\left(\frac{\frac{0.5\cdot[\text{COMB}]}{0.1}+\frac{0.5\cdot[\text{MPH2}]}{0.092}-\frac{0.4\cdot[\text{COM}]}{0.8}+\frac{0.4\cdot[\text{COB}]}{0.25}+\frac{0.4\cdot[\text{"H+\{MAC\}"}]}{0.03}+\frac{0.4\cdot[\text{MP}]}{0.25}}{1+\frac{[\text{COMB}]}{0.1}+\frac{[\text{COB}]}{0.25}+\frac{[\text{MPH2}]}{0.092}+\frac{[\text{COM}]}{0.8}+\frac{[\text{"H+\{MAC\}"}]}{0.03}+\frac{[\text{MP}]}{0.25}}\right)$$

$$+V_{MAC}\cdot\left(\frac{\frac{0.5\cdot[\text{F420H2}]}{0.002}+\frac{0.5\cdot[\text{MP}]}{0.25}-\frac{0.1\cdot[\text{F420}]}{0.043}+\frac{0.1\cdot[\text{MPH2}]}{0.092}+\frac{0.1\cdot[\text{"H+\{MAC\}"}]}{10}}{1+\frac{[\text{F420H2}]}{0.002}+\frac{[\text{MPH2}]}{0.092}+\frac{[\text{MP}]}{0.25}+\frac{[\text{F420}]}{0.043}+\frac{[\text{"H+\{MAC\}"}]}{10}}\right)$$

$$-V_{MAC}\cdot\left(\frac{0.5\cdot[\text{ADP\{MAC\}}]}{2.3225+[\text{ADP\{MAC\}}]}+\frac{0.5\cdot[\text{PO4\{MAC\}}]}{2.3225+[\text{PO4\{MAC\}}]}+\frac{0.5\cdot[\text{"H+\{MAC\}"}]}{2.3225+[\text{"H+\{MAC\}"}]}\right)$$

$$\frac{d([\text{H2O\{MAC\}}]\cdot V_{MAC})}{dt}=-V_{MAC}\cdot\left(\frac{0.5\cdot[\text{CO}]}{0.1553+[\text{CO}]}+\frac{0.5\cdot[\text{H2O\{MAC\}}]}{0.1553+[\text{H2O\{MAC\}}]}+\frac{0.5\cdot[\text{OFD}]}{0.1553+[\text{OFD}]}\right)$$

$$\frac{d([\text{MCOM}]\cdot V_{MAC})}{dt}=+V_{MAC}\cdot\left(\frac{\frac{0.5\cdot[\text{MTHM}]}{0.5}+\frac{0.5\cdot[\text{COM}]}{1}-\frac{0.4\cdot[\text{THM}]}{3.7}+\frac{0.4\cdot[\text{MCOM}]}{0.6}}{1+\frac{[\text{MTHM}]}{0.5}+\frac{[\text{COM}]}{1}+\frac{[\text{MCOM}]}{0.6}+\frac{[\text{THM}]}{3.7}}\right)$$

$$-V_{MAC}\cdot\left(\frac{\frac{0.5\cdot[\text{MCOM}]}{0.6}+\frac{0.5\cdot[\text{COB}]}{0.1}-\frac{0.1\cdot[\text{COMB}]}{1.8}+\frac{0.1\cdot[\text{CH4}]}{0.066}}{1+\frac{[\text{MCOM}]}{0.6}+\frac{[\text{COB}]}{0.1}+\frac{[\text{COMB}]}{1.8}+\frac{[\text{CH4}]}{0.066}}\right)$$

$$\frac{d([\text{MCP}]\cdot V_{MAC})}{dt}=-V_{MAC}\cdot\left(\frac{\frac{0.5\cdot[\text{MCP}]}{0.012}+\frac{0.5\cdot[\text{THM}]}{0.002}-\frac{0.4\cdot[\text{CFP}]}{0.002}+\frac{0.4\cdot[\text{MTHM}]}{0.0756}}{1+\frac{[\text{MCP}]}{0.012}+\frac{[\text{THM}]}{0.002}+\frac{[\text{CFP}]}{0.002}+\frac{[\text{MTHM}]}{0.0756}}\right)$$

$$+V_{MAC}\cdot\left(\frac{\frac{0.5\cdot[ACA\{MAC\}]}{0.28}+\frac{0.5\cdot[CFP]}{0.1}-\frac{0.1\cdot[COA\{MAC\}]}{4.3}+\frac{0.1\cdot[CO]}{0.053}+\frac{0.1\cdot[MCP]}{0.012}}{1+\frac{[ACA\{MAC\}]}{0.28}+\frac{[CFP]}{0.1}+\frac{[COA\{MAC\}]}{4.3}+\frac{[CO]}{0.053}+\frac{[MCP]}{0.012}}\right)$$

$$\frac{d([MP]\cdot V_{MAC})}{dt}=+V_{MAC}\cdot\left(\frac{\frac{0.5\cdot[COMB]}{0.1}+\frac{0.5\cdot[MPH2]}{0.092}-\frac{0.4\cdot[COM]}{0.8}+\frac{0.4\cdot[COB]}{0.25}+\frac{0.4\cdot["H+\{MAC\}"]}{0.03}+\frac{0.4\cdot[MP]}{0.25}}{1+\frac{[COMB]}{0.1}+\frac{[COB]}{0.25}+\frac{[MPH2]}{0.092}+\frac{[COM]}{0.8}+\frac{["H+\{MAC\}"]}{0.03}+\frac{[MP]}{0.25}}\right)$$

$$-V_{MAC}\cdot\left(\frac{\frac{0.5\cdot[F420H2]}{0.002}+\frac{0.5\cdot[MP]}{0.25}-\frac{0.1\cdot[F420]}{0.043}+\frac{0.1\cdot[MPH2]}{0.092}+\frac{0.1\cdot["H+\{MAC\}"]}{10}}{1+\frac{[F420H2]}{0.002}+\frac{[MPH2]}{0.092}+\frac{[MP]}{0.25}+\frac{[F420]}{0.043}+\frac{["H+\{MAC\}"]}{10}}\right)$$

$$\frac{d([MPH2]\cdot V_{MAC})}{dt}=-V_{MAC}\cdot\left(\frac{\frac{0.5\cdot[COMB]}{0.1}+\frac{0.5\cdot[MPH2]}{0.092}-\frac{0.4\cdot[COM]}{0.8}+\frac{0.4\cdot[COB]}{0.25}+\frac{0.4\cdot["H+\{MAC\}"]}{0.03}+\frac{0.4\cdot[MP]}{0.25}}{1+\frac{[COMB]}{0.1}+\frac{[COB]}{0.25}+\frac{[MPH2]}{0.092}+\frac{[COM]}{0.8}+\frac{["H+\{MAC\}"]}{0.03}+\frac{[MP]}{0.25}}\right)$$

$$+V_{MAC}\cdot\left(\frac{\frac{0.5\cdot[F420H2]}{0.002}+\frac{0.5\cdot[MP]}{0.25}-\frac{0.1\cdot[F420]}{0.043}+\frac{0.1\cdot[MPH2]}{0.092}+\frac{0.1\cdot["H+\{MAC\}"]}{10}}{1+\frac{[F420H2]}{0.002}+\frac{[MPH2]}{0.092}+\frac{[MP]}{0.25}+\frac{[F420]}{0.043}+\frac{["H+\{MAC\}"]}{10}}\right)$$

$$\frac{d([MTHM]\cdot V_{MAC})}{dt}=+V_{MAC}\cdot\left(\frac{\frac{0.5\cdot[MCP]}{0.012}+\frac{0.5\cdot[THM]}{0.002}-\frac{0.4\cdot[CFP]}{0.002}+\frac{0.4\cdot[MTHM]}{0.0756}}{1+\frac{[MCP]}{0.012}+\frac{[THM]}{0.002}+\frac{[CFP]}{0.002}+\frac{[MTHM]}{0.0756}}\right)$$

$$-V_{MAC}\cdot\left(\frac{\frac{0.5\cdot[MTHM]}{0.5}+\frac{0.5\cdot[COM]}{1}-\frac{0.4\cdot[THM]}{3.7}+\frac{0.4\cdot[MCOM]}{0.6}}{1+\frac{[MTHM]}{0.5}+\frac{[COM]}{1}+\frac{[MCOM]}{0.6}+\frac{[THM]}{3.7}}\right)$$

$$\frac{d([OFD]\cdot V_{MAC})}{dt}=-V_{MAC}\cdot\left(\frac{0.5\cdot[CO]}{0.1553+[CO]}+\frac{0.5\cdot[H2O\{MAC\}]}{0.1553+[H2O\{MAC\}]}+\frac{0.5\cdot[OFD]}{0.1553+[OFD]}\right)$$

$$\frac{d([PO4\{MAC\}]\cdot V_{MAC})}{dt}=-V_{MAC}\cdot\left(\frac{0.5\cdot[ADP\{MAC\}]}{2.3225+[ADP\{MAC\}]}+\frac{0.5\cdot[PO4\{MAC\}]}{2.3225+[PO4\{MAC\}]}+\frac{0.5\cdot["H+\{MAC\}"]}{2.3225+["H+\{MAC\}"]}\right)$$

$$+V_{MAC}\cdot\left(\frac{\frac{0.5\cdot[ATP\{MAC\}]}{0.133}+\frac{0.5\cdot[ACE\{MAC\}]}{0.258}+\frac{0.5\cdot[COA\{MAC\}]}{0.027}-\frac{0.4\cdot[ADP\{MAC\}]}{1}+\frac{0.4\cdot[PO4\{MAC\}]}{0.742}+\frac{0.4\cdot[ACA\{MAC\}]}{0.28}}{1+\frac{[ATP\{MAC\}]}{0.133}+\frac{[ACE\{MAC\}]}{0.258}+\frac{[COA\{MAC\}]}{0.027}+\frac{[ADP\{MAC\}]}{1}+\frac{[PO4\{MAC\}]}{0.742}+\frac{[ACA\{MAC\}]}{0.28}}\right)$$

$$+V_{MAC}\cdot\left(\frac{\frac{0.5\cdot[ACP\{MAC\}]}{15}+\frac{0.5\cdot[COA\{MAC\}]}{0.034}-\frac{0.4\cdot[ACA\{MAC\}]}{0.023}+\frac{0.4\cdot[PO4\{MAC\}]}{2.8}}{1+\frac{[ACA\{MAC\}]}{0.023}+\frac{[ACP\{MAC\}]}{15}+\frac{[COA\{MAC\}]}{0.034}+\frac{[PO4\{MAC\}]}{2.8}}\right)$$

$$\frac{d([RFD]\cdot V_{MAC})}{dt}=+V_{MAC}\cdot\left(\frac{0.5\cdot[CO]}{0.1553+[CO]}+\frac{0.5\cdot[H2O\{MAC\}]}{0.1553+[H2O\{MAC\}]}+\frac{0.5\cdot[OFD]}{0.1553+[OFD]}\right)$$

$$\frac{d([THM]\cdot V_{MAC})}{dt}=-V_{MAC}\cdot\left(\frac{\frac{0.5\cdot[MCP]}{0.012}+\frac{0.5\cdot[THM]}{0.002}-\frac{0.4\cdot[CFP]}{0.002}+\frac{0.4\cdot[MTHM]}{0.0756}}{1+\frac{[MCP]}{0.012}+\frac{[THM]}{0.002}+\frac{[CFP]}{0.002}+\frac{[MTHM]}{0.0756}}\right)$$

$$+V_{MAC}\cdot\left(\frac{\frac{0.5\cdot[MTHM]}{0.5}+\frac{0.5\cdot[COM]}{1}-\frac{0.4\cdot[THM]}{3.7}+\frac{0.4\cdot[MCOM]}{0.6}}{1+\frac{[MTHM]}{0.5}+\frac{[COM]}{1}+\frac{[MCOM]}{0.6}+\frac{[THM]}{3.7}}\right)$$
